# Subliminal Emotional Faces Elicit Predominantly Right-Lateralized Amygdala Activation: A Systematic Meta-Analysis of fMRI Studies

**DOI:** 10.3389/fnins.2022.868366

**Published:** 2022-07-18

**Authors:** Amelia D. Dahlén, Aphra Schofield, Helgi B. Schiöth, Samantha J. Brooks

**Affiliations:** ^1^Functional Pharmacology and Neuroscience, Department of Surgical Sciences, Uppsala University, Uppsala, Sweden; ^2^Faculty of Health, School of Psychology, Liverpool John Moores University, Liverpool, United Kingdom; ^3^Department of Psychology, School of Human and Community Development, University of Witwatersrand, Johannesburg, South Africa

**Keywords:** subliminal, Activation Likelihood Estimation, emotional faces, amygdala, parahippocampal gyrus

## Abstract

Prior research suggests that conscious face processing occurs preferentially in right hemisphere occipito-parietal regions. However, less is known about brain regions associated with non-conscious processing of faces, and whether a right-hemispheric dominance persists in line with specific affective responses. We aim to review the neural responses systematically, quantitatively, and qualitatively underlying subliminal face processing. PubMed was searched for Functional Magnetic Resonance Imaging (fMRI) publications assessing subliminal emotional face stimuli up to March 2022. Activation Likelihood Estimation (ALE) meta-analyses and narrative reviews were conducted on all studies that met ALE requirements. Risk of bias was assessed using the AXIS tool. In a meta-analysis of all 22 eligible studies (merging clinical and non-clinical populations, whole brain and region of interest analyses), bilateral amygdala activation was reported in the left (x = −19.2, y = 1.5, z = −17.1) in 59% of studies, and in the right (x = 24.4, y = −1.7, z = −17.4) in 68% of studies. In a second meta-analysis of non-clinical participants only (*n* = 18), bilateral amygdala was again reported in the left (x = −18, y = 3.9, z = −18.4) and right (x = 22.8, y = −0.9, z = −17.4) in 56% of studies for both clusters. In a final meta-analysis of whole-brain studies only (n=14), bilateral amygdala was also reported in the left (x = −20.2, y = 2.9, z = −17.2) in 64% of studies, and right (x = 24.2, y = −0.7, z = −17.8) in 71% of studies. The findings suggest that non-consciously detected emotional faces may influence amygdala activation, especially right-lateralized (a higher percentage of convergence in studies), which are integral for pre-conscious affect and long-term memory processing.

## Introduction

Detection of faces and the rapid initiation of appropriate emotional reactions constitutes part of the foundation for smooth social interactions and ultimately our survival. Visual search paradigms, such as “face-in-the-crowd,” highlight how the presence of a face, the direction of attention and whether it poses a potential threat, can be detected within the blink of an eye (Pinkham et al., 2010). This automatic processing is largely attributed to the emotional salience portrayed by a face, whereby expressions of anger, fear and disgust significantly raise our vigilance and give an evolutionary advantage for avoiding harm (Murphy and Zajonc, 1993; Öhman, 2002). By contrast, difficulties with face perception may severely impact social cognitions, e.g., emotion recognition and interpretation of social cues, as seen in autism spectrum disorders and schizophrenia (Kleinhans et al., 2011; Gao et al., 2021).

Emotion processing is suggested to be dichotomized, with one subcortical and subconscious pathway for emotionally salient stimuli, while the other pathway extends into cortex with integration in the amygdala for more detailed perceptual information (LeDoux, 2000; Öhman, 2002). In support of the presence of these two separate pathways, delineating the neural pathways of subconscious face processing is often done with subliminal approaches using temporal backward masking and functional Magnetic Resonance Imaging (fMRI) (Phillips et al., 2004; Kouider et al., 2008). By manipulating the time interval—the Stimulus Onset Asynchrony (SOA)—between the target and the mask, the target stimulus can be interrupted as higher order mechanisms compete with the non-target in object recognition (Enns and Di Lollo, 2000). Despite not being consciously perceived, subliminal stimuli can elicit behavioral and physiological responses, e.g., emotional memories, increased skin conductance, and accompanying neural activity (Sebastiani et al., 2011; Yang et al., 2011). For example, subliminal emotional faces, masked by neutral faces, activate similar brain areas as those presented supraliminally (Prochnow et al., 2013). These regions may include the amygdala, fusiform gyrus, the temporo-parietal junction, and the inferior, dorsolateral, and medial frontal cortex (Prochnow et al., 2013; Freeman et al., 2014).

Lesion and functional neuroimaging studies suggest that a hemispheric lateralization for face processing may exist, with the right hemisphere processing faces holistically and non-consciously, while the left appears to be involved in feature-based processing (Morris et al., 1998; Wada and Yamamoto, 2001; Frässle et al., 2016). Holistic processing is particularly important for face recognition, where key facial features are integrated as a whole rather than being perceived separately (Wang et al., 2012). Emotion processing is also suggested to be lateralized, according to two prevailing hypotheses (Làdavas and Bertini, 2021; Palomero-Gallagher and Amunts, 2021). The right-hemispheric dominance hypothesis postulates that emotions are processed in the right hemisphere independent of their valence. The valence lateralization hypothesis claims that the left is responsible for processing positively valenced stimuli, while the right hemisphere processes negatively valenced stimuli (Palomero-Gallagher and Amunts, 2021). However, it has been debated whether right-hemispheric dominance occurs, and if this occurs independently of conscious awareness and the emotional valence of faces (Fusar-Poli et al., 2009; Gainotti, 2012; Meng et al., 2012).

To answer these questions, and to address the variability of fMRI methodologies—such as differences in participant samples, stimulus presentations, applied contrasts, coordinate systems (e.g., Talairach, MNI, and AFNI), scanner strength and statistical analyses—Activation Likelihood Estimation (ALE) meta-analyses are effective. By analyzing coordinates from individual fMRI studies investigating similar questions, the convergence of foci can be detected with limited confounding effects of varying methodologies, e.g., by weighting foci according to sample size for each study and by using both the statistical significance of individual voxels and a minimum threshold for cluster size (Eickhoff et al., 2012). By doing this, probability maps of neuronal activation can be calculated that lowers the bias introduced by different studies (Kirby and Robinson, 2017). In addition, diagnostic *post-hoc* tests and sub-analyses further improve the risk of bias (Radua and Mataix-Cols, 2012).

Separate ALE approaches have previously been applied to study emotional facial processing and differences between subliminal and supraliminal stimuli (Fusar-Poli et al., 2009; Meneguzzo et al., 2014). In Brooks et al. (2012), significant ALE clusters for subliminally presented emotional faces were found in the right amygdala and the right cerebellum. A right hemisphere specialization for unconscious fear-related stimuli was also discussed in a narrative review by Làdavas and Bertini (2021). However, there have been no ALE meta-analyses since 2012 focusing solely on subliminal emotional face processing and so now additional relevant fMRI studies have been published. As such, we aim to expand on Brooks et al.'s (2012) work by implementing the ALE method to quantitatively (in both whole brain and region of interest analyses) review neural responses underlying subliminal emotional face processing. To further nuance the quantitative ALE review, the goal of the qualitative review is to report emotion-specific neural responses to subliminal faces expressing the basic emotions of happiness, sadness, disgust, fear, surprise, and anger (Ekman, 1984). These six emotions are deemed universal and are suggested to be linked with distinct expressions and physiological changes (Ekman, 1992). Identifying patterns of lateralization may shed light on underlying neural responses to emotional processing of faces, and hint at how facial processing might be altered in developmental and psychiatric disorders that have core deficits in emotion processing (such as autism, schizophrenia, eating disorders).

## Methods

We followed the 10 *Simple Rules for Neuroimaging Meta-analysis* (Müller et al., 2018): (1) Specificity of research question (what are the neural correlates of non-consciously processed emotional faces?). (2) Power of meta-analysis (meta-analyses presented here included *n* = 22, *n* = 18 and an exploratory analysis of more robust whole brain studies *n* = 14, with the suggested limit being *n* = 17–20). (3) Collect and organize data (the data extracted from studies are presented systematically in Table 1). (4) Experiments use the same search coverage (separate ALEs were conducted for all studies and whole brain only). (5) Adjust for multiple contrasts within experiments (no included studies contained multiple studies). (6) Double check data and report (Our data collection, inclusion/exclusion criteria, reported foci and analyses were conducted by AD, AS, and SB and compared). (7) Plan and register analyses (AD, AS, and SB discussed the contrasts to be done and separate analyses. Unfortunately, the protocol was not registered beforehand—it was deemed an update of a previously published study: Brooks et al., 2012). (8) Balance sensitivity and susceptibility for false positives (a more conservative FWE correction was applied to all ALEs). (9) Show diagnostics (the number of studies contributing to the clusters, and the size/ALE value of the cluster were reported. A risk of bias assessment using the AXIS tool was reported). (10) Transparent reporting (detailed search terms, included studies and GingerALE parameters fully described).

**Table 1:** fMRI studies included in ALE meta-analyses and narrative review (*n* = 22).

**Study name**	**Authors**	**Year**	**Subliminal stimuli**	**Control condition**	**Awareness measure**	***N* (F/M)**	**Foci** **(MNI or TAL)**	**fMRI** **analysis**	**All** **studies**	**Non-clinical**	**WB** **only**
Activation of the amygdala and anterior cingulate during non-conscious processing of sad vs. happy faces	Killgore and Yurgelun-Todd	2004	Sad, happy faces	Fixation cross	Objective	12 (12/0)	11 (MNI)	ROI	x	x	
Functional association of the amygdala and ventral prefrontal cortex during cognitive evaluation of facial expressions primed by masked angry faces: an event-related fMRI study	Nomura et al.	2004	Angry faces	Neutral faces (or white blank screen)	Objective	9 (5/4)	4 (TAL)	ROI, WB	x	x	x
Differential neural responses to overt and covert presentations of facial expressions of fear and disgust	Phillips et al.	2004	Fearful, disgusted faces	75% neutral, 25% happy faces	Objective	8 (0/8)	23 (TAL)	WB	x	x	x
Individual differences in trait anxiety predict the response of the basolateral amygdala to unconsciously processed fearful faces	Etkin et al.	2004	Fearful faces	Neutral faces	Objective and subjective	17 (8/9)	9 (MNI)	ROI	x		
A direct brainstem–amygdala–cortical “alarm” system for subliminal signals of fear	Liddell et al.	2005	Fearful faces	Neutral faces	Objective	22 (11/11)	19 (MNI)	ROI, WB	x	x	x
Amygdala–prefrontal dissociation of subliminal and supraliminal fear	Williams et al.	2006	Fearful faces	Neutral faces (or blankstimuli)	Objective	15 (8/7)	9 (MNI)	ROI, WB	x	x	x
Amygdala reactivity predicts automatic negative evaluations for facial emotions	Dannlowski et al.	2007a	Sad, angry, happy faces	Neutral faces (or gray triangle)	Objective	23 (10/11)	9 (MNI)	ROI, WB	x	x	x
Amygdala reactivity to masked negative faces is associated with automatic judgmental bias in major depression: a 3 T fMRI study	Dannlowski et al.	2007b	Sad, angry, happy faces	Neutral faces (or gray triangle)	Objective and subjective	28 (both^*^)	9 (MNI)	ROI, WB	x		x
Neural mechanism of unconscious perception of surprised facial expression	Duan et al.	2010	Surprised, happy faces	Neutral faces	Subjective	18 (13/15)	41 (MNI)	WB	x	x	x
Automatic mood-congruent amygdala responses to masked facial expressions in major depression	Suslow et al.	2010	Happy, sad faces	Neutral faces (or blank screen)	Objective and subjective	56 (27/29)	12 (MNI)	ROI, WB	x		x
Lateralization of amygdala activation in fMRI may depend on phase-encoding polarity	Mathiak et al.	2012	Fearful faces	Neutral faces	Subjective	12 (0/12)	1 (MNI)	ROI	x	x	
Amygdala responses to masked and low spatial frequency fearful faces: a preliminary fMRI study in panic disorder	Ottaviani et al.	2012	Fearful faces	Neutral faces	Objective and subjective	28 (14/14)	2 (TAL)	ROI	x		
The amygdala is involved in affective priming effect for fearful faces	Yang et al.	2012	Fearful faces	Neutral faces	Objective and subjective	27 (13/14)	6 (TAL)	WB	x	x	x
Childhood maltreatment is associated with an automatic negative emotion processing bias in the amygdala	Dannlowski et al.	2013	Sad, happy faces	Neutral faces	Objective and subjective	134 (71/63)	4 (MNI)	ROI, WB	x	x	x
Processing of subliminal facial expressions of emotion: a behavioral and fMRI study	Prochnow et al.	2013	Happy, angry, sad faces	Supraliminal faces	Subjective	18 (13/5)	11 (TAL)	WB	x	x	x
Neural correlates of affective priming effects based on masked facial emotion: an fMRI study	Suslow et al.	2013	Sad, happy, neutral faces	Neutral faces	Objective	110 (58/52)	4 (MNI)	ROI, WB	x	x	x
Trait emotional suppression is associated with increased activation of the rostral anterior cingulate cortex in response to masked angry faces.	Cui et al.	2014	Angry, fearful, happy faces	Neutral faces	Not reported	63 (30/33)	1 (MNI)	ROI	x	x	
Influence of temporal expectations on response priming by subliminal faces	Pichon et al.	2016	Fearful faces	Neutral faces	Objective	30 (15/15)	1 (MNI)	ROI, WB	x	x	x
Effects of electroconvulsive therapy on amygdala function in major depression—a longitudinal functional magnetic resonance imaging study	Redlich et al.	2017	Sad and happy faces	Neutral faces	Not reported	39 (19/20)	2 (MNI)	ROI	x		
Sex differences in neural responses to subliminal sad and happy faces in healthy individuals: implications for depression	Victor et al.	2017	Sad, happy, neutral faces	Neutral faces	Not reported	56 (28/28)	3 (TAL)	ROI	x	x	
Brain response to masked and unmasked facial emotions as a function of implicit and explicit personality self-concept of extraversion.	Suslow et al.	2017	Happy, fearful and disgusted faces	Neutral faces	Not reported	40 (12/28)	6 (MNI)	ROI, WB	x	x	x
Mismatch negativity (MMN) stands at the crossroads between explicit and implicit emotional processing	Chen et al.	2017	Fearful and angry faces	Neutral faces	Objective	30 (14/16)	6 (MNI)	ROI	x	x	

* *Final F/M ratio not reported. MNI, Montreal Neurological Institute; TAL, Talairach*.

### Literature Search

**Figure 1:**
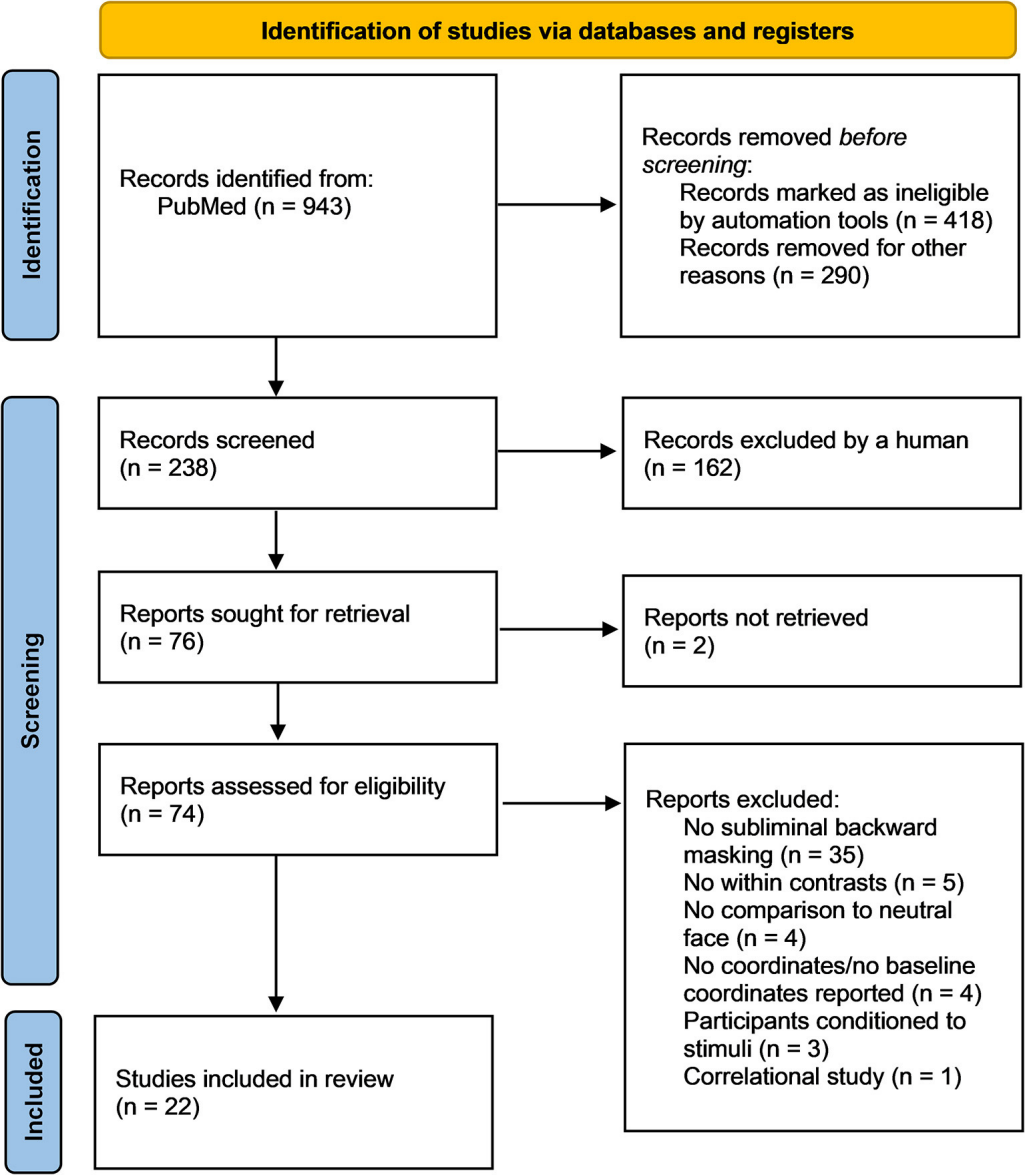
PRISMA flow diagram of the relevant steps for the literature search conducted in PubMed (Page et al., 2021). Out of the initial 943 identified studies, 22 fit the inclusion criteria and were included in the meta-analysis and narrative review. During the identification stage, *n* = 708 studies were removed prior to screening. The *n* = 238 remaining studies were screened and *n* = 76 studies were sought for retrieval. From the *n* = 74 reports assessed for eligibility, *n* = 52 studies were excluded according to exclusion criteria, leaving *n* = 22 studies.

This review followed the Preferred Reporting Items for Systematic Reviews and Meta-Analysis (PRISMA) guidelines (Page et al., 2021; Figure 1). The review was not registered. The PRISMA checklist of recommended items to be reported can be found in Supplementary Table 1. A computerized search using the database PubMed was conducted, covering the periods from 1975 to March 2022 (last search 2022-03-24). Searches were conducted on titles and abstracts containing the following terms (^*^ = truncated): (fMRI OR brain imaging OR functional magnetic resonance imaging) AND (sublim^*^ AND face^*^ AND emotion^*^) OR (unaware^*^ AND face^*^ AND emotion^*^) OR (implicit AND face^*^ AND emotion^*^) OR (unconscious^*^ AND face^*^ AND emotion^*^) OR (non-conscious^*^ AND face^*^ AND emotion^*^) OR (sublim^*^ AND facial^*^ AND emotion^*^) OR (unaware^*^ AND facial^*^ AND emotion^*^) OR (implicit AND facial^*^ AND emotion^*^) OR (unconscious^*^ AND facial^*^ AND emotion^*^) OR (non-conscious^*^ AND facial^*^ AND emotion^*^). Additional filters were utilized to refine the search further; these included: Adults (18+), human participants, English language, no reviews, no case studies, and no meta-analyses.

### Inclusion/Exclusion Criteria

To prevent selection bias, the PRISMA recommendations for systematic literature analysis have been followed and two authors (AD and AS) selected studies based on the broad search terms. Studies were included if they: (a) used task-based fMRI, (b) were conducted using subliminal/non-conscious presentation of faces using backward masking, (c) included human faces of any gender, race, age, and demonstrating any emotion vs. a neutral face or neutral image, (d) reported the neural activation or deactivation co-ordinates in Montreal Neurological Institute (MNI) or Talairach space, (e) assessed brain regional activation and deactivation using contrast analyses and not regression models, (f) were published in English, and (g) were published in a peer-reviewed journal article. Out of the initial 943 identified studies, 22 fit the inclusion criteria and were included in the meta-analysis and the narrative review (Figure 1). During the identification stage, *n* = 708 studies were removed prior to screening. The *n* = 238 remaining studies were screened and *n* = 76 studies were sought for retrieval.

### Selected Studies

Of the 74 publications assessed for eligibility, 52 did not meet the inclusion criteria (Figure 1, Supplementary Table 2). Studies were excluded if they did not provide within-subjects contrasts comparing subliminal emotional face stimuli with subliminal neutral stimuli, if the participants were conditioned to the stimuli, if the participants were children, if the participants were made aware of the subliminal presentations before testing and if MNI or Talairach coordinates were not reported. Thus, 22 publications remained and were included in the meta-analysis and the narrative review. Risk of bias in individual studies was assessed using the AXIS appraisal tool (Supplementary Table 3). Of these 22 studies, eight were region of interest (ROI) studies, four were whole brain studies, and 10 reported both ROI and whole brain results. The total number of participants was *n* = 796 (50:50 women:men). In the first meta-analysis, all 22 studies (both ROI and whole brain) were included regardless of participant population; the second meta-analysis used only 18 studies (again both ROI and whole brain) that did not include clinical populations; the third meta-analysis excluded the 8 ROI studies (to prevent biasing of ALE results), and examined the 14 whole brain studies as an exploratory analysis. Across the reviewed studies, the subliminal emotional face stimuli expressed fear, happiness, sadness, anger, surprise, and disgust. Control conditions included neutral faces, fixation cross, blank screen, no-face stimulus (a gray rectangle), visual noise masks of scrambled faces and non-emotional images (e.g., tomato). See Table 1 for details of included studies in both the quantitative and qualitative analysis. The analyzed contrasts from each study are reported in Supplementary Table 4.

### fMRI Methods

Functional Magnetic Resonance Imaging (fMRI) is a non-invasive brain imaging technique that indirectly measures neuronal activation *via* Blood Oxygen Level Dependency (BOLD). fMRI studies utilize either a whole brain or a ROI technique to measure BOLD. Using whole brain, significant activation clusters are localized by examining voxels across the global space. Conversely, ROI analyses use a mask to include or remove a brain area/areas from statistical analysis. ROI analyses are less favorable to include in ALE analyses as they tend to inflate the significance of the meta-analysis. ROI analyses differ from small volume correction (SVC) in terms of to what this test is applied and the subsequent output. For example, ROIs return a single *t*/*F*-value for the whole ROI, given that the test is applied to an average signal of all voxels. Conversely, in the SVC procedure, the test is applied to all voxels (voxel-wise) and therefore the output is a volumetric statistical map with *t*/*F*-values for each voxel within the small volume. ROI analyses were reported by eight studies included in the current meta-analyses, and so additional analyses were conducted to examine whether these ROI studies biased the overall findings.

### Quantitative Data Analyses—ALE

BrainMap GingerALE version 3.0.2 software (Laird et al., 2005; Turkeltaub et al., 2012) was used to complete the ALE analyses described above, with the updated version of the ALE approach (Eickhoff et al., 2009). Foci were extracted from publications examining neural responses to subliminal face perception, checked by two researchers (AS, SJB). Papers that reported coordinates in standard Talairach space were converted into MNI using the GingerALE software. Text files were subsequently created, listing the study names, number of subjects and a list of the foci (MNI coordinates) associated with neural activation to subliminal stimuli. Specifically, three separate ALE analyses were conducted: (a) utilizing all 22 publications incorporating both whole brain and ROI, and including all types of participant (e.g., clinical and healthy controls); (b) 18 publications that only examined subliminal face processing in healthy controls; (c) an exploratory examination including 14 publications of only healthy controls in whole brain studies (excluding ROI studies). Text files of foci were cross-checked by two researchers as above and are provided as Supplementary Material (Data Sheet).

ALE is a statistical modeling technique examining variance between and within fMRI studies using the total foci coordinates reported in each study to build a three-dimensional Gaussian kernel, enabling a modeled activation (MA) threshold map for each study (Eickhoff et al., 2012). Differences in foci positions can be a consequence of between-study variance, e.g., in templates used or heterogeneity of participants, and as such these two main issues are considered in the parameters of the kernel. This is done by weighting the foci reported by the number of participants in each study. Finally, MA maps for each study are combined for each separate meta-analysis, creating an experimental ALE map. This is tested against the null hypothesis that there is random variation in relation to the spatial orientation of neural activation for the specific meta-analysis (e.g., subliminal presentation of faces), but that the within-study variation is fixed. A random effects model is employed by the ALE analysis technique, which assumes a higher than chance likelihood of consensus between different experiments, but not in relation to activation variance within each study. The null distribution map is permuted by the number of studies that constitute each meta-analysis. To correct for multiple comparisons, we used a threshold of *p* < 0.05 False Discovery Rate (FDR), and chose a minimum cluster size of 100 mm^3^, in accordance with our recent ALE publications on this subject (Brooks et al., 2012; Meneguzzo et al., 2014), and we also used a more conservative (as opposed to dilated) kernel threshold under ALE preferences (Eickhoff et al., 2012). We used an anatomical image overlay program called Mango (http://ric.uthscsa.edu/mango) to illustrate the results of our meta-analyses. GingerALE employs the term “contributing studies,” to describe studies that are located within the boundaries of ALE cluster. However, this does not discount other studies that might be located near these boundaries but outside of the cluster, which could have also contributed to it.

## Results—Quantitative/ALE-Meta-Analysis

**Figure 2:**
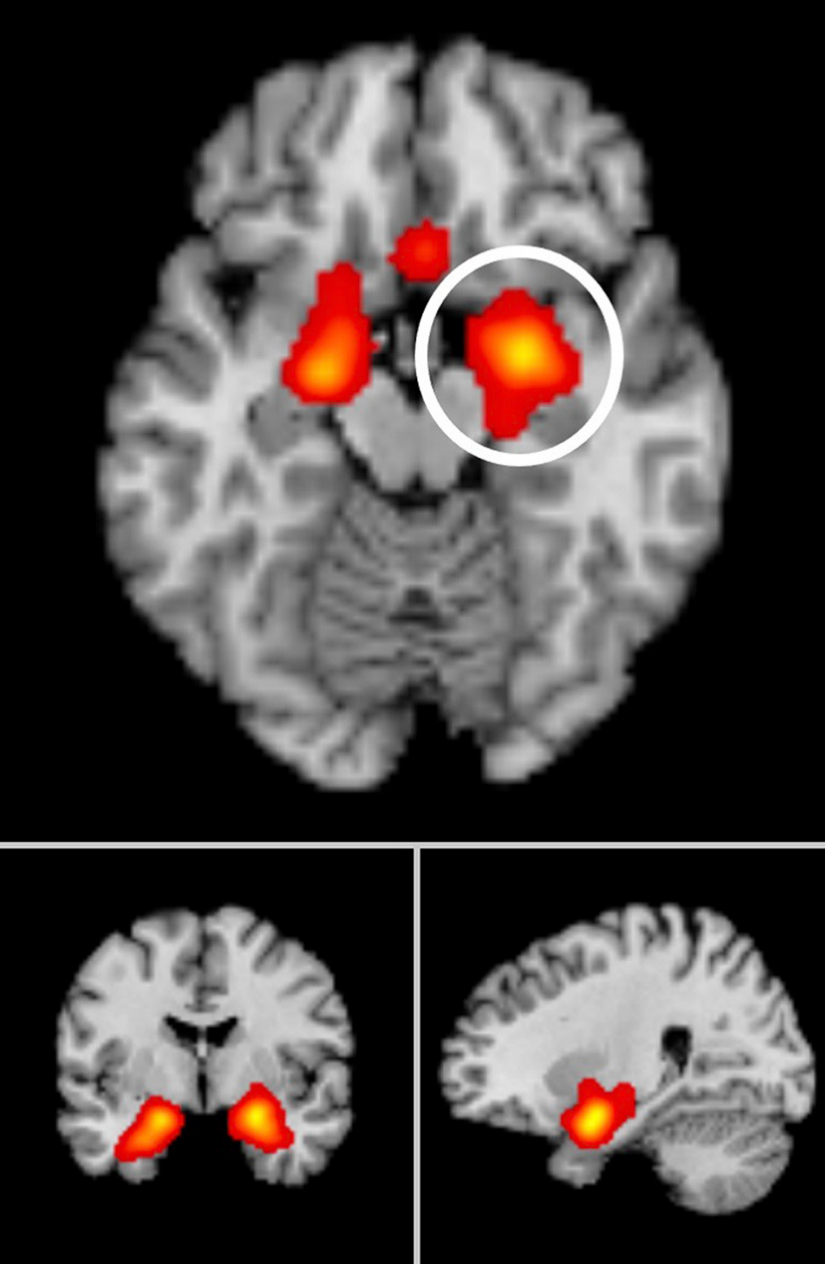
Significant ALE cluster maxima of neural activation to subliminal emotional face stimuli surviving FDR correction, *p* < 0.05 for multiple comparisons, cluster size >100 mm^3^. Montreal Neurological Institute (MNI) coordinates are given. Meta-analysis 1 peak clusters: left amygdala (x = −19.1, y = 1.5, z = −17.1), right amygdala (x = 24.4, y = −1.7, z = −17.4). Meta-analysis 2 peak clusters: left amygdala (x = −18, y = 3.9, z = −18.4), right amygdala (x = 22.8, y = −0.9, z = −17.4). Meta-analysis 3 peak clusters: left amygdala (x = −20.2, y = 2.9, z = −18.4), right amygdala (x = 22.8, y = −0.9, z = −17.4).

The significant clusters including those that were significant at the cluster-level family-wise error (FWE) correction, with a more conservative (smaller) mask threshold, are reported in Table 2 for each meta-analysis, and in Figure 2.

**Table 2:** Clusters of statistically significant activation.

**ALE analysis**	**Anatomical label**	**Peak voxel co-ordinates**			**Cluster size** **(mm^**3**^)**	**ALE value** **(x 10^**−2**^)**	**No of contributing experiments**	
		**x**	**y**	**z**				**%**
All studies (*n* = 22)	1 L Amygdala	−19.2	1.5	−17.1	16,984	0.0406	13	59
	2 R Amygdala	24.4	−1.7	−17.4	13,400	0.0393	15	68
Non-clinical ROI & WB (*n* = 18)	1 L Amygdala	−18	3.9	−18.4	13,216	0.0242	10	56
	2 R Amygdala	22.8	−0.9	−17.4	10,232	0.0296	10	56
WB Only (*n* = 14)	1 L Amygdala	−20.2	2.9	−17.2	15,888	0.0251	9	64
	2 R Amygdala	24.2	−0.7	−17.8	13,688	0.0244	10	71

### Meta-Analysis One: Significant ALE Clusters From All Studies

From 193 foci, 835 subjects, and 22 separate studies (24 experiments), the ALE analysis revealed two significant clusters that survived FWE correction. The first cluster was centered in the left amygdala (x = −19.2, y = 1.5, z = −17.1). The second cluster was centered in the right amygdala (x = 24.4, y = −1.7, z = −17.4).

### Meta-Analysis Two: Significant ALE Clusters From Non-clinical Samples

From 168 foci, 647 subjects and 18 separate studies (18 experiments), two clusters survived the FWE correction. The first cluster was centered in the left amygdala (x = −18, y = 3.9, z = −18.4). The second cluster was centered in the right amygdala (x = 22.8, y = −0.9, z = −17.4).

### Meta-Analysis Three: Significant ALE Clusters From Whole Brain Studies Only

We also ran a separate exploratory analysis of the whole brain studies alone, excluding any studies which only conducted ROI analyses. From 144 foci, 541 subjects, and 14 studies, the ALE analysis revealed two clusters which survived FWE correction and the predefined cluster criterion. Cluster one was centered in the left amygdala (x = −20.2, y = 2.9, z = −17.2). Cluster two was centered in the right amygdala (x = 24.2, y = −0.7, z = −17.8).

Of note, the highest percentage of contributing studies was 71% for activation in the right amygdala in whole brain studies only (*n* = 10), and while this was a small number of studies, this finding was corroborated by 68% of all studies reporting right amygdala activation to subliminal emotional faces (*n* = 15).

## Results—Qualitative/Narrative Review

**Figure 3:**
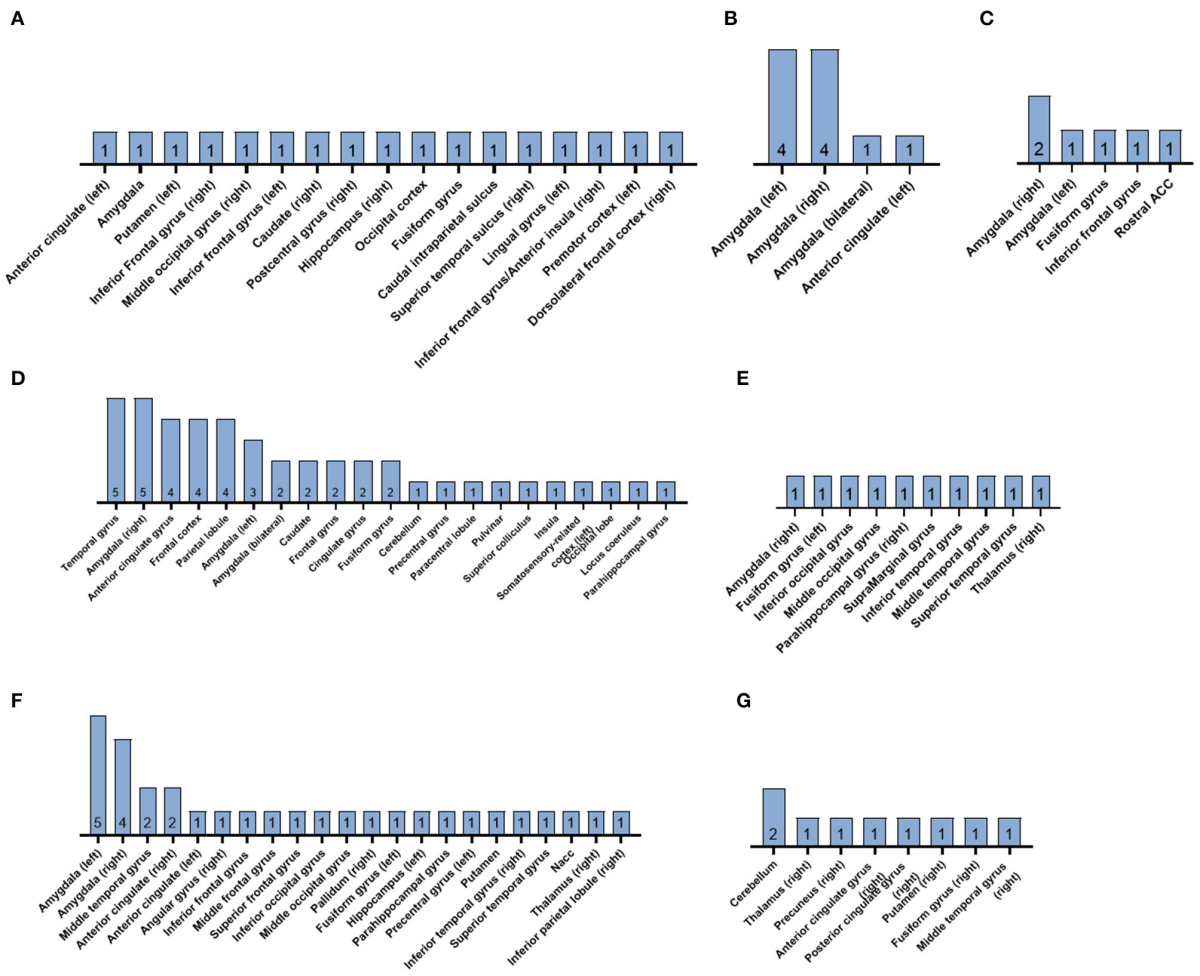
Summary diagrams of the reported clusters of significant brain activation in response to subliminal emotional stimuli in the reviewed fMRI studies. Subliminally presented faces expressed **(A)** mixed emotional expressions, **(B)** sadness, **(C)** anger, **(D)** fear, **(E)** surprise, **(F)** happiness, and **(G)** disgust. The number within each bar indicates the *n* of the studies. ACC, anterior cingulate cortex; NAcc, nucleus accumbens.

A summary of the reported clusters of significant brain activation in response to subliminal emotional stimuli for each of the 22 included studies is presented in Figure 3.

### Emotional Faces: Anger, Happiness, and Sadness Combined

Prochnow et al. (2013) presented facial expressions of anger, happiness, and sadness supraliminally (400 ms) or subliminally (40 ms). Both supraliminal and subliminal conditions of emotional faces elicited activation in the occipital cortex (OCC), fusiform gyrus, and caudal intra-parietal sulcus (cIPS), in comparison to baseline conditions with neutral face masks. Supraliminal emotional faces also activated the right superior temporal sulcus (STS), the right posterior cingulate cortex, the right superior colliculi, the right inferior frontal gyrus (IFG) bordering the anterior insula, and the right dorsolateral prefrontal cortex (DLPFC). For the subliminal emotional faces there was an increase in activation along the posterior right STS, the left premotor cortex area 6, and in the right DLPFC (Figure 3). Control objects (supraliminal non-emotional images, e.g., chair, followed by unemotional adjective pairs) were associated with bilateral activation in the cerebellum, occipital cortex, dorsal paracingulate cortex/anterior cingulate cortex (ACC), DLFC, and left premotor area 6.

### Fearful Faces

Ten studies with healthy adult samples investigated the subliminal processing of fearful faces in comparison to neutral faces. Liddell et al. (2005) found that masked (16.7 ms) presentations of fearful facial expressions evoked significantly increased activity in the left superior colliculus, left pulvinar, bilateral amygdalae, and bilateral anterior cingulate. The right amygdala showed a larger cluster of activation than the left. Whole brain analysis confirmed the significant activity observed in the left ventral anterior cingulate seen in the ROI analysis. Whole brain analysis also demonstrated significant responses to subliminal fear in fronto-temporal and somatosensory-related cortices.

In Pichon et al. (2016), subliminal face presentation was conducted using both forward masking and backward masking (66 ms after the prime) with visual noise masks (scrambled face stimuli). The subliminal faces elicited activations in the bilateral occipital face area (OFA), right fusiform face area (FFA), bilateral inferior frontal gyrus (IFG BA45 and BA47), but no activation in the amygdala. Fearful face primes resulted in left parahippocampal gyrus activation, which was enhanced when the target faces appeared at the expected rather than the unexpected time. However, significant changes in activation in the amygdala and FFA were not found. Unlike the influence of temporal attention, the gender congruence of the prime and target face did not influence the parahippocampal gyrus activation.

When comparing subliminal and supraliminal presentations of fearful facial expressions, Williams et al. (2006) detected significant activity in the left amygdala, the left dorsal ACC and in the medial prefrontal cortex (MPFC) extending to the right hemisphere in supraliminal fear conditions relative to neutral conditions. Subliminal fear conditions, relative to neutral, elicited significant activity in the bilateral amygdala and ventral right MPFC, extending into the ventral ACC. Whole brain analysis between-conditions confirmed that supraliminal fear had significantly greater responses relative to subliminal fear in DLPFC and visual regions. In contrast, subliminal fear elicited comparatively greater responses in the right hypothalamus and the right ventral ACC.

Yang et al. (2012) used encoding and retrieval phases to investigate the affective priming effect for fearful faces. Backward masked fearful or neutral faces were presented during the encoding and during the retrieval participants judged the fearful or neutral expression of the target face. Participants who were unaware the subliminal priming had stronger activation in the left amygdala, while participants who were aware of the priming had stronger activity in the left prefrontal cortex, left occipital region and the left fusiform gyrus. Moreover, unaware participants primed with fearful faces (vs. neutral) produced greater activity in the right amygdala and the right pulvinar. In aware participants, fearful faces generated stronger activity in the left amygdala, right fusiform gyrus and the left STS. They also found a congruency effect whereby congruent faces (fearful–fearful) elicited weaker amygdala activation than incongruent faces (neutral–fearful).

The supraliminal (179 ms) and subliminal fearful faces (30 ms) were presented to male participants in Phillips et al. (2004). Both conditions activated regions of visual processing: the left precuneus, the bilateral superior and the right middle temporal gyri for subliminal fear, and bilateral superior and the right middle temporal and lingual gyri and precuneus for supraliminal fear. Both supraliminal and subliminal fear activated the left inferior frontal and right anterior cingulate gyri, bilateral inferior parietal lobules, and the right cerebellum. Voxels of significant activation in the right amygdala were detected in the supraliminal fear condition, but not in the subliminal condition. Subliminal fear elicited activation in the left caudate nucleus. Moreover, the left inferior frontal gyrus, the left inferior parietal lobule and bilateral cerebellum showed significantly greater mean power of response to subliminal fear than supraliminal presentations of fear.

Masked fear (33 ms) in Etkin et al. (2004) elicited significant activation in the right basolateral amygdala, which was also found to be correlated with anxious traits in the healthy adult sample. Non-masked fear (200 ms) did not lead to basolateral amygdala activation, but instead right dorsal amygdala activation, which was not correlated to anxiousness. Chen et al. (2017) observed right amygdala activation in response to masked fearful faces vs. neutral faces, while non-masked fearful faces vs. neutral faces activated the left amygdala.

Eleven out of 12 male participants in Mathiak et al. (2012) showed amygdala responses to fearful faces, whereby seven subjects had bilateral activation, three had left-lateralized activation and one right-lateralized activation. However, volume-corrected thresholds for masked fearful stimuli did not generate significant amygdala activity. Nor did masked fearful faces in Cui et al. (2014) generate significant activation in their predefined ROIs (bilateral amygdala, insula, ACC, mPFC, and orbitofrontal cortex) or in the ROIs of Suslow et al. (2017) (amygdala, thalamus, caudate nuclei, and putamen).

### Happy Faces

Of the reviewed fMRI studies with healthy adult participants, eight included masked happy faces in their subliminal paradigms. Dannlowski et al. (2013) found significant activations in the bilateral amygdala for subliminally presented happy faces (33 ms) followed by a neutral target face (467 ms). Likewise, adult women in Killgore and Yurgelun-Todd (2004) showed significant activation within the left and right amygdala following happy faces masked by neutral faces. In addition, they detected clusters of elevated activation in the bilateral anterior cingulate gyri. The observed right amygdala activation and bilateral anterior cingulate gyri activation during the masked happiness condition were found to be significantly greater than in the masked sadness condition. Suslow et al. (2017) reported clusters of significant activation in response to masked happy faces in the right inferior parietal lobule to right middle temporal regions and in the right thalamus. These clusters were also positively correlated to implicit extraversion measures.

Happy faces presented subliminally in Duan et al. (2010), elicited increased neural activity in the left amygdala, anterior cingulate, left inferior frontal/orbito-frontal gyrus, and right inferior temporal gyrus, in comparison to neutral faces. When contrasting masked happy faces with masked surprised faces, the happy faces were associated with increased activation of the left posterior cingulate gyrus, middle frontal gyrus, and middle temporal gyrus. Masked presentation of happy faces may also activate the bilateral nucleus accumbens (NAcc), as demonstrated in Suslow et al. (2013). The right NAcc activation was also found to have a significant positive correlation with the affective priming score of the neutral target face, however no significant priming based on happy faces was established.

In Victor et al. (2017), females showed reduced BOLD responses to masked happy vs. masked neutral faces in the left amygdala, the right subgenual anterior cingulate cortex (sgACC), and the right pregenual ACC (pgACC), relative to men. When amygdala activity was recorded in response to masked faces with happy, sad and angry expressions using fMRI in Dannlowski et al. (2007a), no significant effect of gender was observed for any of the investigated emotions. Regarding happy faces vs. neutral faces, no significant bilateral amygdala activity was found during subliminal presentation. However, in comparison to the no-face baseline, subliminal happy faces elicited activations in the right amygdala. Masked happy faces in Cui et al. (2014) did not generate significant activation in their predefined ROIs (bilateral amygdala, insula, ACC, mPFC, and orbitofrontal cortex).

### Sad Faces

Five of the studies examined the brain activation patterns elicited by subliminal faces with sad expressions in healthy adults. Dannlowski et al. (2013) found that sad faces presented subliminally (33 ms) followed by a neutral target face (467 ms) significantly activated the bilateral amygdala. Suslow et al. (2013) also found significant bilateral amygdala activation following the masked presentation of sad faces. This amygdala activation had significant positive correlations with the affective priming scores given to the neutral target faces. In addition, whole brain analysis revealed a positive correlation between medial frontal gyrus activation and the affective priming score based on sad faces, which was significantly higher than the positive correlation between amygdala activation and affective priming scores.

The subliminal presentation of sad faces in Dannlowski et al. (2007a) yielded significant bilateral amygdala activity, in comparison to both neural faces and the no-face baseline. In addition, greater amygdala responses to masked sad sadness were associated with more negative bias scores given to neutral target faces. However, in the female sample of Killgore and Yurgelun-Todd (2004), masked sad faces only generated significantly greater activation within the right amygdala, whereas the left amygdala did not show significant activation. When compared to the masked happiness condition, the masked sadness did not have any suprathreshold voxels within the ROIs. Female participants in Victor et al. (2017), showed elevated BOLD responses to viewing masked sad faces vs. masked happy faces in the right hippocampus and the bilateral sgACC, compared to male participants.

### Angry Faces

The processing of subliminal angry facial expressions was investigated in four of the reviewed studies (Figure 3). Significantly increased bilateral amygdala activity was seen for masked angry faces, in comparison to neutral faces, in Dannlowski et al. (2007a). However, the elevated amygdala activity following presentation of angry faces was not significantly higher than the no face baseline condition. Similar to the sad faces, the stronger amygdala activation in response to masked angry faces was associated with more negative bias scores given to neutral target faces. Cui et al. (2014) found a cluster of activation in the rostral ACC in response to masked angry faces. Rostral ACC activation was also correlated with higher scores on measures of anger and anxiety. In Nomura et al. (2004), the anger prime, neutral prime and control conditions all produced activation in the right fusiform gyrus. Right amygdala activation was only detected in the anger prime condition. Moreover, the responses in the right amygdala had a significant negative correlation with activity in the right inferior frontal gyrus within the angry face prime condition. A significant positive correlation was found between the rate of judgment of anger of the target face and activation intensity in the right amygdala. In Chen et al. (2017) neither subliminal nor supraliminal angry faces elicited amygdala activation in comparison to neutral faces.

### Surprised Faces

One of the reviewed fMRI studies focused on the neural responses to surprised faces presented below conscious awareness. In comparison to masked neutral faces, masked surprised faces were associated with higher activity in the right parahippocampal gyrus, left fusiform gyrus, right amygdala, and right thalamus (Duan et al., 2010). Above threshold activation was also detected in clusters within the occipital lobe and temporal lobe. When comparing masked surprised faces with masked happy faces, surprise showed increased activation in the bilateral amygdala, parahippocampal gyrus, fusiform gyrus, and cingulate gyrus. Clusters of suprathreshold activation were also present in the cerebellum, inferior frontal gyrus, temporal, and occipital lobes.

### Disgusted Faces

Disgusted faces were investigated by Phillips et al. (2004) using subliminal (30 ms) and supraliminal (170 ms) presentations in a male sample. Both conditions activated regions of visual processing: the right fusiform gyrus, precuneus, and middle temporal gyrus in response to subliminal, and the right precuneus, left middle occipital, bilateral lingual, right superior, and left middle temporal gyri in response to supraliminal faces of disgust. Significantly activated voxels were also detected in the bilateral posterior cingulate and right anterior cingulate gyri and the left cerebellum. In addition, supraliminal disgust activated bilateral insulae, while subliminal disgust did not. Subliminal presentations of disgust activated the right thalamus and putamen. Suslow et al. (2017) also used backwardly masked disgusted faces (17 ms) in healthy adult men and women. The subliminal faces of disgust generated clusters in the bilateral anterior cerebellum and the cerebellal lingual.

### Studies With Clinical Samples

The participants in Dannlowski et al. (2007b) suffered from acute major depression. They described clusters of significant activation in both the left and right amygdala for subliminal sad faces vs. neutral faces, and for subliminal angry faces vs. neutral faces. In contrast, the subliminally presented happy faces did not elicit significant changes in brain activation in comparison to neutral faces. No activation by masked happy faces could be detected in comparison with the neutral face baseline. However, when compared to a no-face baseline, masked happy faces were associated with activations in the right amygdala. In participants with an acute major depressive episode, amygdala responses to masked sad faces were greater than responses to masked happy faces (Suslow et al., 2010). The bilateral amygdala responses to subliminal faces expressing sadness were also significantly greater than the bilateral amygdala responses seen in healthy control subjects shown subliminal sad faces. In contrast, the healthy control group had greater bilateral amygdala responses to happy compared with sad faces and compared with the depressed group's bilateral amygdala responses to happy faces.

Redlich et al. (2017) used fMRI to investigate the automatic processing of subliminally presented emotional faces in participants with major depressive disorder (MDD) before and after treatment with electroconvulsive therapy (ECT), in a control sample of MDD patients treated with pharmacotherapy and in a healthy control sample. Both MDD groups had increased bilateral amygdala activity to sad faces compared to the healthy control group at baseline. There was no significant difference for the happy face condition between the groups at baseline. After ~6 weeks with brief pulse ECT treatment or pharmacotherapy, both MDD groups displayed a reduced bilateral amygdala activity in reaction to subliminal sad faces. The pharmacotherapy MDD group also demonstrated a significant increase in amygdala activity to subliminal happy faces, but the ECT MDD group did not. Moreover, no significant differences in amygdala activity in response to subliminal emotional faces compared to controls was detected after 6 weeks with brief pulse ECT treatment or pharmacotherapy, indicating a normalization of the MDD patient groups.

In Ottaviani et al. (2012), healthy control participants showed bilateral amygdala activation in response to backward masking of fearful faces, while participants with panic disorder did not. Masked fearful faces elicited higher amygdala responses than masked neutral faces.

## Discussion

The present ALE meta-analysis explored neural activation in response to subliminal emotional faces during fMRI and found bilateral clusters of activation in the amygdala across all studies, as well as those that only examined healthy participants, and those only using whole brain analyses. Meta-analysis one, combining ROI studies with both clinical and non-clinical samples demonstrated bilateral amygdala activation, with a higher percentage of studies reporting right amygdala activation. Meta-analysis two, which only examined non-clinical samples also reported bilateral amygdala, converging equally on left and right. Finally, meta-analysis three presented only whole-brain studies, and again demonstrated bilateral amygdala activation, most often reported by studies on the right side. Moreover, the narrative review highlighted how the degree of positive or negative affect of the subliminally presented faces may elicit different patterns of neural activation. In particular, increased activation was observed across corticolimbic regions, but predominantly visual cortex areas in response to general subliminal emotional faces. Fearful and happy faces tended to activate bilateral limbic regions (Figure 3). Of the studies examining sad and angry faces, a potentially right-specific amygdala/fusiform area response was detected. Surprise and disgust appeared to activate subcortical regions in the few studies that examined these emotions.

### Right-Lateralized Amygdala Activation During Subliminal Conditions in Non-clinical ROI Studies and Whole Brain Studies

According to the affective circumplex model, emotions contain two independent dimensions: valence (the negative or positive nature of the emotion) and arousal (the heightened physiological activity caused by the emotion), which activate distinct neural pathways (Posner et al., 2005; Gerber et al., 2008). The subcortically located amygdala has long been considered a key region for processing faces and their emotions, as lesions within the amygdala can severely impair emotion recognition (Adolphs et al., 1994). The amygdala has also been suggested to encode the different dimensions of emotion in a lateralized manner (Wang et al., 2017). The right amygdala may preferentially subserve the uncertainty of a stimulus' valence, while the left may decode the emotional arousal of a stimulus (Gläscher and Adolphs, 2003; Colibazzi et al., 2010; Wang et al., 2017). When contrasting subliminal and supraliminal emotion processing, the right amygdala has been implicated in automatic responses to emotion, while the left amygdala is activated during conscious reflection following emotional stimuli (Dyck et al., 2011; Pantazatos et al., 2012). A potential explanatory model could therefore involve a right amygdala-driven automatic and global emotional response, followed by a left amygdala-driven differentiated emotional reaction which incorporates information on arousal to further guide cognition and behavior (Gläscher and Adolphs, 2003; Pantazatos et al., 2012).

The present findings of significant ALE clusters within the right amygdala, based on the reviewed non-clinical ROI studies and the whole brain studies, slightly differ from the bilateral activation following subliminal emotional faces found in Brooks et al. (2012). However, while Brooks et al. (2012) argued for more robust right amygdala activation, prior imaging studies have also reported elevated left amygdala activation in response to emotionally arousing stimuli such as facial expressions (Morris et al., 1996; Lane et al., 1997; Vytal and Hamann, 2010). More specifically, left amygdala activation has been associated with fearful facial expressions (Hardee et al., 2008). However, of the 10 reviewed studies using fearful face masks, the reports of significant amygdala activation were mixed. Liddell et al. (2005) and Yang et al. (2012) reported bilateral amygdala activity with greater right lateralization. Etkin et al. (2004) and Chen et al. (2017) also reported right amygdala activation. Conversely, Williams et al. (2006) detected significant left amygdala activation, while Phillips et al. (2004), Mathiak et al. (2012) Suslow et al. (2013), Cui et al. (2014), and Pichon et al. (2016), did not detect significantly altered amygdala activity in either hemisphere for the subliminal fear conditions. The significant right amygdala clusters detected at group level may therefore be due to the different emotional face expressions, e.g., happiness and sadness, used across the included non-clinical ROI studies. Likewise, the experimental designs of the 14 studies included in the whole brain analysis used subliminal faces with varying emotional valence which could underlie variance in amygdala activation.

### Right Parahippocampal Gyrus Activation During Subliminal Conditions in Non-clinical ROI Studies and Whole Brain Studies

Two of the conducted ALE analyses, including whole brain studies analyzed separately, revealed significant right-lateralized activation which could be interpreted as merging onto the parahippocampal gyrus, which is located anteromedially to the amygdala and adjacent to the hippocampus. In addition to the amygdala-centered network, the hippocampus-centered network plays a pivotal role in emotion processing (Palomero-Gallagher and Amunts, 2021). By integrating emotional information from the amygdala, *via* the ACC, the hippocampal complex modulates the consolidation and retrieval of emotional memories (Carlson et al., 2013; Bian et al., 2019). The parahippocampal gyrus takes part in the limbic system and its activation together with the amygdala is in accordance with previous ALE meta-analyses on implicit emotion processing (Li et al., 2010; Shi et al., 2013), Interestingly, Shi et al. (2013) found that masking tasks were associated with parahippocampal gyrus and amygdala activation, while inattention tasks of implicit emotional processing preferentially activated the fusiform gyrus. The different activation patterns elicited by masking tasks and inattention tasks, respectively, suggest that the former targets early stages of emotional processing while the latter captures a later stage of pre-attentive emotional processing (Shi et al., 2013). The results provide support for the ability of emotional faces to rapidly activate subcortical brain regions, before cortical structures are recruited to evaluate the emotional stimulus and coordinate an appropriate response (LeDoux, 2000; Öhman, 2002).

Paradigms using supraliminal emotional stimuli (e.g., emotionally evocative sentences), have detected strong associations between elevated amygdala and parahippocampal activity and arousal ratings (Colibazzi et al., 2010; Meneguzzo et al., 2014). Indeed, emotional arousal is a well-established mediator in memory consolidation *via* the amygdalo-hippocampal-parahippocampal network (Dolcos et al., 2004). The current findings of clusters with significant activation within the right amygdala and parahippocampal gyrus for subliminal emotional faces, suggests how non-conscious stimuli may influence memory formation and thereby guide behaviors by facilitating the recognition of familiar faces and the emotions they may be expressing. For example, attentional biases toward masked fearful faces may predispose individuals to anxiety or exacerbate sensations of threat, despite being in a safe environment (Carlson et al., 2013). This may in turn lead to a generalization of contextual fear, as seen in posttraumatic stress disorder, which can severely impact everyday life (Bian et al., 2019).

Anger has previously been associated with activations of the right parahippocampal gyrus (Vytal and Hamann, 2010). Here, Nomura et al. (2004) and Dannlowski et al. (2007a) did not report significant changes in parahippocampal gyrus activity during masked anger conditions. However, surprised faces were associated with higher activity in the right parahippocampal gyrus (Duan et al., 2010), whereas fearful face primes generated left parahippocampal gyrus activation (Pichon et al., 2016). Victor et al. (2017) detected gender differences with female participants demonstrating elevated BOLD responses to masked sad faces vs. masked happy faces in the right hippocampus, compared to male participants. Functional interaction studies have proposed distinct amygdala neural signatures associated with supraliminal facial expressions of different emotions (Diano et al., 2017). As the current meta-analysis did not conduct separate ALE analyses for each subliminally presented emotion, no conclusions can be drawn regarding the valence lateralization hypothesis, where the left processes positively valenced stimuli and the right processes negatively valenced stimuli (Palomero-Gallagher and Amunts, 2021). However, the indications of right-lateralized amygdala and parahippocampal gyrus activity from the reviewed ROI studies and whole brain studies, regardless of emotional valence, suggests that the right-hemispheric dominance hypothesis may apply to subliminal processing as well (Palomero-Gallagher and Amunts, 2021).

### Right Striatum Activation During Subliminal Conditions in Non-clinical ROI Studies and Whole Brain Studies

Some studies reported in the narrative review demonstrate increased activation to subliminal emotional faces in the striatum. The striatum is comprised of the caudate, putamen, nucleus accumbens, and is primarily known for its role in voluntary motor control (Báez-Mendoza and Schultz, 2013). Neuroimaging work has highlighted the striatum's involvement in integrating social information and reward processes (Báez-Mendoza and Schultz, 2013). Moreover, the ventral striatum has been found to be more sensitive to subliminally presented faces in comparison to supraliminally presented faces (Ito et al., 2015).

During emotion identification, the striatum has been suggested to act in opposition to the amygdala (Satterthwaite et al., 2011). For example, while the amygdala may preferentially react to threatening angry or fearful faces, the striatum may be tuned to detect non-threatening sad or happy faces (Satterthwaite et al., 2011). While two of the studies in the current review reported increased putamen and nucleus accumbens activation in response to masked happy faces (Duan et al., 2010; Suslow et al., 2013), three reported putamen and caudate activation in response to masked negative emotions (Phillips et al., 2004; Liddell et al., 2005; Dannlowski et al., 2007a). Despite the mixed emotional valences, the right striatum activation seen in the reviewed studies, suggests that subliminal face processing is not only important for threat perception but also for drawing our attention to cues which could lead to social rewards, such as social inclusion (Báez-Mendoza and Schultz, 2013).

### Clinical Samples of Major Depressive Disorder and Panic Disorder

Mood disorders are often characterized by emotional dysfunctions which manifest as abnormal affective processing and behaviors, such as altered reward appraisal and negative attentional biases (Diekhof et al., 2008). When it comes to face processing, depressed patients may have trouble recognizing neutral faces and have been shown to wrongfully ascribe sadness to supraliminal neutral expressions (Leppänen et al., 2004). Prior work has attributed these depression-related tendencies to amygdala hyperactivity, but it is not yet established whether this aberrant processing should be regarded as a causal factor or a clinical manifestation of depression (Stuhrmann et al., 2011). Intriguingly, a hyperactive amygdala state can also be detected during the subliminal presentations of sad faces, as indicated by the reviewed studies with MDD patients (Dannlowski et al., 2007b; Suslow et al., 2010; Redlich et al., 2017). The MDD participants also had greater amygdala responses to masked sad faces in comparison to masked happy faces, while the opposite was seen for the healthy controls (Suslow et al., 2010).

A previous ALE meta-analysis comparing MDD patients and healthy volunteers also found different brain activation patterns depending on the stimulus valence (emotional faces or words; Groenewold et al., 2013). Negative stimuli elicited greater activation in the right amygdala, left striatum, dorsal anterior cingulate and parahippocampal areas in the MDD participants. The same brain regions were activated in response to positive stimuli, but to a lesser extent than the healthy controls (Groenewold et al., 2013). The opposing neural activation patterns, with the amygdala highly tuned to negative information (Murray et al., 2011), and lower activity in the ventral striatum tuned to positive information (Diekhof et al., 2008), support a negative attention bias in depression where negative stimuli may be preferentially processed (Gotlib et al., 2004). Given that subliminal faces expressing sadness may also elicit elevated amygdala responses in MDD patients, as seen in Suslow et al. (2010) and Redlich et al. (2017), it is emphasized how depression-relevant information may be subconsciously processed and is therefore difficult to change. However, Redlich et al. (2017) demonstrated how amygdala activity in response to subliminal emotional faces can be normalized in MDD patients following 6 weeks of ECT treatment or pharmacotherapy. This highlights the possibilities of altering dysfunctional emotional face processing in mood disorders.

In contrast to the above findings from MDD patients, participants with panic disorder did not show amygdala activation in response to backward masking of fearful faces in Ottaviani et al. (2012). Although the masked fearful faces did elicit higher amygdala responses than the masked neutral faces. Patients with panic disorder suffer from recurrent panic attacks, accompanying physical symptoms such as an increased pulse or chest pain, and anticipatory anxiety (Sobanski and Wagner, 2017). Supraliminal presentations of emotional faces have previously generated increased amygdala activation in participants with anxiety disorders when viewing fearful faces relative to happy faces, compared with healthy controls (Fonzo et al., 2015). Furthermore, anxiety-prone individuals have been shown to have significantly higher bilateral amygdala and insula activation to emotional faces (Stein et al., 2007). A hyperactive insula has also been associated with panic disorder diagnoses (Fonzo et al., 2015). The insula is associated with interoceptive processes which signal internal body states (Sobanski and Wagner, 2017). Elevated insula activity may therefore reflect an increased sensitivity to distressing body sensations in individuals with panic disorder (Fonzo et al., 2015). As Ottaviani et al. (2012) relied on a ROI-based analysis for the amygdala only, no results were reported for the insula.

### Strengths and Limitations

A major strength of the current study is the use of ALE methodology which addresses the difficulties with meta-analyzing fMRI studies, due to the different statistical contrasts used in creating neural activation images. An additional strength is that the right amygdala activation (extending into the right parahippocampal gyrus) remained significant when the analysis was re-run without the ROI studies, which strengthens the findings. However, the ALE approach does not account for the intensity of the BOLD signal reported in each study. It is also possible that differences in the fMRI set-ups and participant instructions could have influenced the results. The results may also have been affected by differences in the sample demographics. For example, Killgore and Yurgelun-Todd (2004) only tested women, while Phillips et al. (2004) and Mathiak et al. (2012) only included men. However, there were not enough studies to conduct an ALE to examine gender differences.

The studies in the current meta-analysis all used masking tasks which have been proven useful for examining the early stages of implicit emotional processing (Shi et al., 2013). Of note, binocular rivalry and interocular suppression approaches can also be used in the study of emotional face processing, but this meta-analysis only included studies using masking tasks (Alpers and Gerdes, 2007; Yang and Yeh, 2018). The studies presented the masks for 40 ms or shorter, which is deemed sufficient to prevent the conscious detection of the prime stimulus (Esteves and Öhman, 1993). However, detection of non-conscious primes can occur below 40 ms (Szczepanowski and Pessoa, 2007), which is why it is important to pair the masking tasks with a subsequent detection task to ensure that the participants did not perceive the masked stimulus. Both subjective and objective criteria to test the potential awareness of the prime were used across the reviewed studies (Table 1). Some studies relied solely on subjective reports of visual awareness (e.g., Duan et al., 2010; Mathiak et al., 2012; Prochnow et al., 2013). Other studies used objective discrimination thresholds where unaware perception was defined as by chance performance in forced-choice detection tasks (e.g., Liddell et al., 2005; Ottaviani et al., 2012; Pichon et al., 2016). As information about the non-conscious stimulus may only be accessible for a limited time, it may fade before visual awareness can be self-reported and lead to incorrect assumptions being made for the subliminal experimental conditions. Therefore, objective criteria may be more suitable as a determinant (Pessoa et al., 2006; Pichon et al., 2016). However, running sub-analyses for studies using objective or subjective criteria was unfortunately not possible due to the limited number of whole brain studies in this ALE.

## Conclusions

This study is, to our knowledge, the first ALE meta-analysis focusing on fMRI studies of subliminal emotional face processing. Clusters of significant activation were detected in bilateral amygdala, with most study convergence in the right amygdala, in response to non-consciously presented multi-valenced images of faces. Given the integral role of the amygdalo-hippocampal-parahippocampal network in memory formation, it is compelling to consider how these brain regions may be activated without conscious awareness. An impaired ability to interpret emotional facial cues may be particularly worrisome for individuals suffering from mental health conditions. Future fMRI studies of non-consciously presented, multi-valenced affective stimuli will be important in delineating the implicated neural pathways in both functional and dysfunctional states.

## Data Availability Statement

The original contributions presented in the study are included in the article/Supplementary Material, further inquiries can be directed to the corresponding author/s.

## Author Contributions

AD: manuscript preparation, investigation, formal analysis, and data curation. AS: investigation, formal analysis, data curation, and editing. HS: supervision. SB: conceptualization, formal analysis, resources, supervision, and write-up and editing. All authors contributed to manuscript revision, read, and approved the submitted version.

## Funding

HS was supported by the Swedish Research Council. The funders had no role in the design, analysis, and write-up or decision to submit for publication.

## Conflict of Interest

The authors declare that the research was conducted in the absence of any commercial or financial relationships that could be construed as a potential conflict of interest.

## Publisher's Note

All claims expressed in this article are solely those of the authors and do not necessarily represent those of their affiliated organizations, or those of the publisher, the editors and the reviewers. Any product that may be evaluated in this article, or claim that may be made by its manufacturer, is not guaranteed or endorsed by the publisher.
