# Supplementary material for: Subliminal Emotional Faces Elicit Predominantly Right-Lateralized Amygdala Activation: A Systematic Meta-Analysis of fMRI Studies
Source: Front Neurosci. 2022 Jul 18;16:868366. doi: 10.3389/fnins.2022.868366 (PMC9339677; doi:10.3389/fnins.2022.868366)
Supplement: Supplementary file 1 [file Data_Sheet_1.DOCX]

Text files for meta-analyses:

**Meta-analysis 1**

Mask:

Reference Space = MNI

Dimensions = 77x96x79

Number of within-brain voxels = 229781

Mask Size = More Conservative (Smaller)

Foci:

Coordinate System = MNI

File of foci coordinates = Txtfile1_June2022.txt

Number of foci = 193

Number of experiments = 24

Total number of subjects = 835

ALE - Random Effects, Turkeltaub Non-Additive (HBM, 2012):

File of ALE voxels = Txtfile1_June2022_ALE.nii

FWHM minimum value = 8.540284452174179

FWHM median value = 9.137264222884284

FWHM maximum value = 10.362276299774612

Minimum ALE score = 6.8842035E-35

Maximum ALE score = 0.0405653

P Values: Eickhoff (HBM, 2009)

File of P values = Txtfile1_June2022_P.nii

Minimum P value = 4.444317E-14

Thresholding:

Threshold Method = Cluster-level Inference

Thresholding Value = 0.05

Thresholding Permutations = 1000

Cluster-Forming Method = Uncorrected P value

Cluster-Forming Value = 0.05

Volume > Threshold = 30384 mm^3

Chosen min. cluster size = 6432 mm^3

Thresholded ALE image = Txtfile1_June2022_C05_1k_ALE.nii

Cluster Analysis:

#1: 16984 mm^3 from (-40,-18,-36) to (8,28,10) centered at (-19.2,1.5,-17.1) with 8 peaks

with a max value of 0.0406 ALE, 4.444317E-14 P, 7.46 Z at (-22,-6,-18)

Labels: (Gray Matter only)

Hemisphere: 97.3% Left Cerebrum, 2.4% Right Cerebrum

Lobe: 56.5% Sub-lobar, 35.3% Limbic Lobe, 7.5% Frontal Lobe

Gyrus: 37.8% Lentiform Nucleus, 22.2% Parahippocampal Gyrus, 11.8% Caudate, 8.4% Uncus, 5.7% Thalamus, 4.5% Anterior Cingulate, 4.1% Inferior Frontal Gyrus, 3.7% Subcallosal Gyrus

Cell Type: 26.4% Putamen, 16.8% Amygdala, 11.8% Caudate Head, 11.6% Brodmann area 34, 8.7% Lateral Globus Pallidus, 5.1% Brodmann area 28, 4.6% Brodmann area 47, 3% Brodmann area 25, 2.2% Medial Globus Pallidus, 1.6% Brodmann area 32

#2: 13400 mm^3 from (10,-20,-32) to (40,14,0) centered at (24.4,-1.7,-17.4) with 4 peaks

with a max value of 0.0393 ALE, 1.3512793E-13 P, 7.31 Z at (24,0,-18)

Labels: (Gray Matter only)

Hemisphere: 100% Right Cerebrum

Lobe: 57% Sub-lobar, 37.9% Limbic Lobe, 4.6% Frontal Lobe

Gyrus: 54.8% Lentiform Nucleus, 28.6% Parahippocampal Gyrus, 9.1% Uncus, 3.1% Subcallosal Gyrus, 1.8% Inferior Frontal Gyrus

Cell Type: 32% Putamen, 21% Amygdala, 16.5% Lateral Globus Pallidus, 16% Brodmann area 34, 6.3% Medial Globus Pallidus, 4.9% Brodmann area 28, 1.8% Brodmann area 47

Experiment Table:

[ 1 0 0 0 2 2 1 2 2 4 4 3 1 1 2 2 0 2 0 0 0 2 0 0 ]

[ 1 2 1 0 1 0 1 2 2 5 5 2 0 1 1 2 0 1 0 0 1 0 0 1 ]

Contributors to cluster #1

1 foci from //Killgore 2004

2 foci from //Lidell 2005

2 foci from //Lidell 2005

1 foci from //Williams 2006

2 foci from //Williams 2006

2 foci from //Dannlowski 2007a

4 foci from //Dannlowski 2007b

4 foci from //Duan 2010

3 foci from //Suslow 2010

1 foci from //Mathiak 2012

1 foci from //Ottaviani 2012

2 foci from //Yang 2012

2 foci from //Dannlowski 2013

2 foci from //Suslow 2013

2 foci from //Victor 2017

Contributors to cluster #2

1 foci from //Killgore 2004

2 foci from //Nomura 2004

1 foci from //Phillips 2004

1 foci from //Lidell 2005

1 foci from //Williams 2006

2 foci from //Williams 2006

2 foci from //Dannlowski 2007a

5 foci from //Dannlowski 2007b

5 foci from //Duan 2010

2 foci from //Suslow 2010

1 foci from //Ottaviani 2012

1 foci from //Yang 2012

2 foci from //Dannlowski 2013

1 foci from //Suslow 2013

1 foci from //Redlich 2017

1 foci from //Chen 2017

**Meta-analysis 2:**

Mask:

Reference Space = MNI

Dimensions = 77x96x79

Number of within-brain voxels = 229781

Mask Size = More Conservative (Smaller)

Foci:

Coordinate System = MNI

File of foci coordinates = Txtfile2.txt

Number of foci = 168

Number of experiments = 18

Total number of subjects = 647

ALE - Random Effects, Turkeltaub Non-Additive (HBM, 2012):

File of ALE voxels = Txtfile2_ALE.nii

FWHM minimum value = 8.540284452174179

FWHM median value = 9.168897450170782

FWHM maximum value = 10.362276299774612

Minimum ALE score = 6.8842035E-35

Maximum ALE score = 0.029571217

P Values: Eickhoff (HBM, 2009)

File of P values = Txtfile2_P.nii

Minimum P value = 1.4313502E-10

Thresholding:

Threshold Method = Cluster-level Inference

Thresholding Value = 0.05

Thresholding Permutations = 1000

Cluster-Forming Method = Uncorrected P value

Cluster-Forming Value = 0.05

Volume > Threshold = 23448 mm^3

Chosen min. cluster size = 5408 mm^3

Thresholded ALE image = Txtfile2_C05_1k_ALE.nii

Cluster Analysis:

#1: 13216 mm^3 from (-40,-12,-36) to (8,28,0) centered at (-18,3.9,-18.4) with 7 peaks

with a max value of 0.0242 ALE, 1.4361596E-8 P, 5.55 Z at (-22,-6,-18)

Labels: (Gray Matter only)

Hemisphere: 95.9% Left Cerebrum, 4.1% Right Cerebrum

Lobe: 47.4% Sub-lobar, 47% Limbic Lobe, 5.3% Frontal Lobe

Gyrus: 32% Lentiform Nucleus, 28.8% Parahippocampal Gyrus, 14% Caudate, 10.8% Uncus, 7.1% Anterior Cingulate, 5.3% Subcallosal Gyrus

Cell Type: 28% Putamen, 21.3% Amygdala, 14.8% Brodmann area 34, 14% Caudate Head, 6.6% Brodmann area 28, 4.2% Brodmann area 25, 3.7% Lateral Globus Pallidus, 2.9% Brodmann area 32, 1.6% Brodmann area 24, 1.2% Brodmann area 47

#2: 10232 mm^3 from (10,-20,-30) to (32,14,0) centered at (22.8,-.9,-17.4) with 3 peaks

with a max value of 0.0296 ALE, 1.4313502E-10 P, 6.31 Z at (24,2,-20)

Labels: (Gray Matter only)

Hemisphere: 100% Right Cerebrum

Lobe: 53.8% Sub-lobar, 41.1% Limbic Lobe, 5.1% Frontal Lobe

Gyrus: 51.5% Lentiform Nucleus, 32% Parahippocampal Gyrus, 8.8% Uncus, 3.7% Subcallosal Gyrus, 1.7% Inferior Frontal Gyrus

Cell Type: 33.2% Putamen, 22.2% Amygdala, 19.4% Brodmann area 34, 13.8% Lateral Globus Pallidus, 4.5% Medial Globus Pallidus, 4.3% Brodmann area 28, 1.7% Brodmann area 47

Experiment Table:

[ 1 0 0 0 4 3 2 4 1 2 2 0 1 0 0 1 0 0 ]

[ 1 2 0 0 1 3 2 5 0 1 2 0 1 0 0 0 0 1 ]

Contributors to cluster #1

1 foci from //Killgore 2004

4 foci from //Lidell 2005

3 foci from //Williams 2006

2 foci from //Dannlowski 2007

4 foci from //Duan 2010

1 foci from //Mathiak 2012

2 foci from //Yang 2012

2 foci from //Danlowski 2013

1 foci from //Suslow 2013

1 foci from //Victor 2017

Contributors to cluster #2

1 foci from //Killgore 2004

2 foci from //Nomura 2004

1 foci from //Lidell 2005

3 foci from //Williams 2006

2 foci from //Dannlowski 2007

5 foci from //Duan 2010

1 foci from //Yang 2012

2 foci from //Danlowski 2013

1 foci from //Suslow 2013

1 foci from //Chen 2017

**Meta-analysis 3:**

Mask:

Reference Space = MNI

Dimensions = 77x96x79

Number of within-brain voxels = 229781

Mask Size = More Conservative (Smaller)

Foci:

Coordinate System = MNI

File of foci coordinates = Txtfile3.txt

Number of foci = 144

Number of experiments = 14

Total number of subjects = 541

ALE - Random Effects, Turkeltaub Non-Additive (HBM, 2012):

File of ALE voxels = Txtfile3_ALE.nii

FWHM minimum value = 8.540284452174179

FWHM median value = 9.137264222884284

FWHM maximum value = 10.362276299774612

Minimum ALE score = 4.0387465E-38

Maximum ALE score = 0.025106806

P Values: Eickhoff (HBM, 2009)

File of P values = Txtfile3_P.nii

Minimum P value = 4.1526325E-9

Thresholding:

Threshold Method = Cluster-level Inference

Thresholding Value = 0.05

Thresholding Permutations = 1000

Cluster-Forming Method = Uncorrected P value

Cluster-Forming Value = 0.05

Volume > Threshold = 29576 mm^3

Chosen min. cluster size = 7496 mm^3

Thresholded ALE image = Txtfile3_C05_1k_ALE.nii

Cluster Analysis:

#1: 15888 mm^3 from (-42,-12,-36) to (14,28,2) centered at (-20.2,2.9,-17.2) with 10 peaks

with a max value of 0.0251 ALE, 4.1526325E-9 P, 5.76 Z at (-22,-6,-18)

Labels: (Gray Matter only)

Hemisphere: 89.3% Left Cerebrum, 10.7% Right Cerebrum

Lobe: 55.8% Sub-lobar, 35.9% Limbic Lobe, 8.2% Frontal Lobe

Gyrus: 33.4% Lentiform Nucleus, 25.1% Parahippocampal Gyrus, 19.3% Caudate, 7.5% Uncus, 5.5% Inferior Frontal Gyrus, 3.3% Anterior Cingulate, 2.7% Subcallosal Gyrus, 1.5% Claustrum

Cell Type: 27% Putamen, 18.5% Amygdala, 18.1% Caudate Head, 12.1% Brodmann area 34, 6.3% Brodmann area 47, 5.2% Lateral Globus Pallidus, 4.4% Brodmann area 28, 1.7% Brodmann area 25, 1.2% Caudate Body, 1% Medial Globus Pallidus

#2: 13688 mm^3 from (8,-22,-32) to (40,16,0) centered at (24.2,-.7,-17.8) with 5 peaks

with a max value of 0.0244 ALE, 7.506976E-9 P, 5.66 Z at (24,2,-22)

Labels: (Gray Matter only)

Hemisphere: 100% Right Cerebrum

Lobe: 52.9% Sub-lobar, 37.4% Limbic Lobe, 9% Frontal Lobe

Gyrus: 50.8% Lentiform Nucleus, 27.8% Parahippocampal Gyrus, 9.4% Uncus, 6.1% Inferior Frontal Gyrus, 3.2% Subcallosal Gyrus

Cell Type: 34.6% Putamen, 19.1% Amygdala, 16.2% Brodmann area 34, 11.7% Lateral Globus Pallidus, 6% Brodmann area 47, 5.7% Brodmann area 28, 4.6% Medial Globus Pallidus

Experiment Table:

[ 0 0 3 2 2 4 4 1 2 2 0 2 0 0 ]

[ 2 1 0 2 2 5 5 1 1 2 0 1 0 0 ]

Contributors to cluster #1

3 foci from //Lidell 2005

2 foci from //Williams 2006

2 foci from //Dannlowski 2007

4 foci from //Dannlowski 2007

4 foci from //Duan 2010

1 foci from //Suslow 2010

2 foci from //Yang 2012

2 foci from //Dannlowski 2013

2 foci from //Suslow 2013

Contributors to cluster #2

2 foci from //Nomura 2004

1 foci from //Phillips 2004

2 foci from //Williams 2006

2 foci from //Dannlowski 2007

5 foci from //Dannlowski 2007

5 foci from //Duan 2010

1 foci from //Suslow 2010

1 foci from //Yang 2012

2 foci from //Dannlowski 2013

1 foci from //Suslow 2013
