# Supplementary material for: Subliminal Emotional Faces Elicit Predominantly Right-Lateralized Amygdala Activation: A Systematic Meta-Analysis of fMRI Studies
Source: Front Neurosci. 2022 Jul 18;16:868366. doi: 10.3389/fnins.2022.868366 (PMC9339677; doi:10.3389/fnins.2022.868366)
Supplement: Supplementary file 3 [file Table_2.docx]

| \| **Supplementary Table S2:** Rejected papers for ALE meta-analyses and narrative review \| \| \| \| \| --- \| --- \| --- \| --- \| \| **Study name** \| **Authors** \| **Year** \| **Rejection Reason** \| \| Brain structures activated by overt and covert emotional visual stimuli. \| Sabatini \| 2009 \| Participants conditioned to stimuli \| \| [Target visibility and visual awareness modulate amygdala responses to fearful faces.](https://pubmed.ncbi.nlm.nih.gov/15930371/) \| Pessoa \| 2006 \| Participants conditioned to stimuli \| \| Implicitly and explicitly assessed anxiety: No relationships with recognition of and brain response to facial emotions. \| Suslow \| 2019 \| Participants conditioned to stimuli \| \| [Amygdala excitability to subliminally presented emotional faces distinguishes unipolar and bipolar depression: an fMRI and pattern classification study.](https://pubmed.ncbi.nlm.nih.gov/24038516/) \| Grotegerd \| 2014 \| No within contrasts \| \| [Amygdala Reactivity to Emotional Faces in the Prediction of General and Medication-Specific Responses to Antidepressant Treatment in the Randomized iSPOT-D Trial.](https://pubmed.ncbi.nlm.nih.gov/25824424/) \| Williams \| 2015 \| No within contrasts \| \| [Amygdala Activation and Connectivity to Emotional Processing Distinguishes Asymptomatic Patients With Bipolar Disorders and Unipolar Depression.](https://pubmed.ncbi.nlm.nih.gov/30343134/) \| Korgaonkar \| 2019 \| No within contrasts \| \| Distinct neural mechanisms of emotional processing in prolonged grief disorder. \| Bryant \| 2021 \| No within contrasts \| \| [Using standardized fMRI protocols to identify patterns of prefrontal circuit dysregulation that are common and specific to cognitive and emotional tasks in major depressive disorder: first wave results from the iSPOT-D study.](https://pubmed.ncbi.nlm.nih.gov/23303059/) \| Korgaonkar \| 2013 \| No within contrasts \| \| [Hypervigilance for fear after basolateral amygdala damage in humans.](https://pubmed.ncbi.nlm.nih.gov/22832959/) \| Terburg \| 2012 \| No coordinates reported \| \| Behavioral and neural correlates of visual emotion discrimination and empathy in mild cognitive impairment. \| Pernigo \| 2015 \| No coordinates for faces \| \| Real-Time Functional Magnetic Resonance Imaging Amygdala Neurofeedback Changes Positive Information Processing in Major Depressive Disorder. \| Young \| 2017 \| No coordinates for baseline \| \| [Changes in the neural correlates of implicit emotional face processing during antidepressant treatment in major depressive disorder.](https://pubmed.ncbi.nlm.nih.gov/23809145/) \| Victor et al. \| 2013 \| No baseline pre-treatment coordinates given \| \| [Emotional intelligence is associated with reduced insula responses to masked angry faces.](https://pubmed.ncbi.nlm.nih.gov/26053697/) \| Alkozei & Killgore \| 2015 \| Correlation \| \| [Mood-congruent amygdala responses to subliminally presented facial expressions in major depression: associations with anhedonia.](https://pubmed.ncbi.nlm.nih.gov/23171695/) \| Stuhrmann \| 2013 \| no comparison to neutral \| \| [Masked presentations of emotional facial expressions modulate amygdala activity without explicit knowledge.](https://pubmed.ncbi.nlm.nih.gov/9412517/) \| Whalen \| 1998 \| no comparison to neutral \| \| [Amygdala activation to masked happy facial expressions.](https://pubmed.ncbi.nlm.nih.gov/19958569/) \| Juruena \| 2010 \| no comparison to neutral \| \| [Emotion specific modulation of automatic amygdala responses by 5-HTTLPR genotype.](https://pubmed.ncbi.nlm.nih.gov/19962442/) \| Dannlowski et al. \| 2010 \| no comparison to neutral \| \| [Preliminary report on the association between pulvinar volume and the ability to detect backward-masked facial features.](https://pubmed.ncbi.nlm.nih.gov/29097112/) \| Kim \| 2019 \| Eye whites pooled with facial expressions. \| \| Amygdala activation and facial expressions: explicit emotion discrimination versus implicit emotion processing. \| Habel \| 2007 \| No subliminal masking \| \| Facial expression primes and implicit regulation of negative emotion. \| Yoon \| 2015 \| No subliminal masking \| \| Proactive engagement of cognitive control modulates implicit approach-avoidance bias. \| Harlé \| 2020 \| No subliminal masking \| \| An event related functional magnetic resonance imaging study of facial emotion processing in Asperger syndrome. \| Deeley \| 2007 \| No subliminal masking \| \| Age-related differences in brain activity during implicit and explicit processing of fearful facial expressions. \| Zsoldos \| 2016 \| No subliminal masking \| \| Affective blindsight relies on low spatial frequencies. \| Burra \| 2019 \| No subliminal masking \| \| Facial fear processing and psychotic symptoms in schizophrenia: functional magnetic resonance imaging study. \| Michalopoulou \| 2008 \| No subliminal masking \| \| Functional similarity of facial emotion processing between people with a first episode of psychosis and healthy subjects. \| Villalta-Gil \| 2013 \| No subliminal masking \| \| Neural circuitry of emotion regulation: Effects of appraisal, attention, and cortisol administration. \| Ma \| 2017 \| No subliminal masking \| \| Cerebral differences in explicit and implicit emotional processing--an fMRI study. \| Scheuerecker \| 2007 \| No subliminal masking \| \| Differential Modulation of Effective Connectivity in the Brain's Extended Face Processing System by Fearful and Sad Facial Expressions. \| Jamieson \| 2021 \| No subliminal masking \| \| Psychopathy and functional magnetic resonance imaging blood oxygenation level-dependent responses to emotional faces in violent patients with schizophrenia. \| Dolan \| 2009 \| No subliminal masking \| \| Dissociable brain correlates for depression, anxiety, dissociation, and somatization in depersonalization-derealization disorder. \| Lemche \| 2016 \| No subliminal masking \| \| Attenuated responses to emotional expressions in women with generalized anxiety disorder. \| Palm \| 2011 \| No subliminal masking \| \| Affect and neural activity in women with PTSD during a task of emotional interference. \| Brown \| 2016 \| No subliminal masking \| \| Cognitive vulnerability and implicit emotional processing: imbalance in frontolimbic brain areas? \| Groenewold \| 2015 \| No subliminal masking \| \| Visual cortical regions show sufficient test-retest reliability while salience regions are unreliable during emotional face processing. \| McDermott \| 2020 \| No subliminal masking \| \| Neural correlates of anxiety sensitivity in panic disorder: A functional magnetic resonance imaging study. \| Poletti \| 2015 \| No subliminal masking \| \| Dissociable patterns of medial prefrontal and amygdala activity to face identity versus emotion in bipolar disorder. \| Keener \| 2012 \| No subliminal masking \| \| Neural basis of implicit memory for socio-emotional information in schizophrenia. \| Schwartz \| 2013 \| No subliminal masking \| \| Neuronal correlates of emotional processing in patients with major depression. \| Frodl \| 2009 \| No subliminal masking \| \| Blunted neural response to implicit negative facial affect in anorexia nervosa. \| Leppanen \| 2017 \| No subliminal masking \| \| Effect of task conditions on brain responses to threatening faces in social phobics: an event-related functional magnetic resonance imaging study. \| Straube \| 2004 \| No subliminal masking \| \| Explicit and implicit neural mechanisms for processing of social information from facial expressions: a functional magnetic resonance imaging study. \| Critchley \| 2000 \| No subliminal masking \| \| Functional neural correlates of emotional expression processing deficits in behavioural variant frontotemporal dementia. \| Virani \| 2013 \| No subliminal masking \| \| Cerebral and autonomic responses to emotional facial expressions in depersonalisation disorder. \| Lemche \| 2008 \| No subliminal masking \| \| Effect of specific psychotherapy for chronic depression on neural responses to emotional faces. \| Klein \| 2014 \| No subliminal masking \| \| Amygdala hypersensitivity in response to emotional faces in Tourette's patients. \| Neuner \| 2010 \| No subliminal masking \| \| Temporal unpredictability of a stimulus sequence and the processing of neutral and emotional stimuli. \| Koppe \| 2015 \| No subliminal masking \| \| Increased BOLD signal in the fusiform gyrus during implicit emotion processing in anorexia nervosa. \| Fonville \| 2013 \| No subliminal masking \| \| Influence of Familial Risk for Depression on Cortico-Limbic Connectivity During Implicit Emotional Processing. \| Wackerhagen \| 2017 \| No subliminal masking \| \| Prefrontal cortical response to emotional faces in individuals with major depressive disorder in remission. \| Kerestes \| 2012 \| No subliminal masking \| \| Neural responses associated with positive and negative emotion processing in patients with left versus right temporal lobe epilepsy. \| Batut \| 2006 \| No subliminal masking \| \| Developmental differences in neuronal engagement during implicit encoding of emotional faces: an event-related fMRI study. \| Nelson \| 2003 \| No subliminal masking \| |
| --- | --- | --- | --- | --- | --- | --- | --- | --- | --- | --- | --- | --- | --- | --- | --- | --- | --- | --- | --- | --- | --- | --- | --- | --- | --- | --- | --- | --- | --- | --- | --- | --- | --- | --- | --- | --- | --- | --- | --- | --- | --- | --- | --- | --- | --- | --- | --- | --- | --- | --- | --- | --- | --- | --- | --- | --- | --- | --- | --- | --- | --- | --- | --- | --- | --- | --- | --- | --- | --- | --- | --- | --- | --- | --- | --- | --- | --- | --- | --- | --- | --- | --- | --- | --- | --- | --- | --- | --- | --- | --- | --- | --- | --- | --- | --- | --- | --- | --- | --- | --- | --- | --- | --- | --- | --- | --- | --- | --- | --- | --- | --- | --- | --- | --- | --- | --- | --- | --- | --- | --- | --- | --- | --- | --- | --- | --- | --- | --- | --- | --- | --- | --- | --- | --- | --- | --- | --- | --- | --- | --- | --- | --- | --- | --- | --- | --- | --- | --- | --- | --- | --- | --- | --- | --- | --- | --- | --- | --- | --- | --- | --- | --- | --- | --- | --- | --- | --- | --- | --- | --- | --- | --- | --- | --- | --- | --- | --- | --- | --- | --- | --- | --- | --- | --- | --- | --- | --- | --- | --- | --- | --- | --- | --- | --- | --- | --- | --- | --- | --- | --- | --- | --- | --- | --- | --- | --- | --- | --- | --- | --- | --- | --- | --- | --- | --- | --- |
