# Supplementary material for: Subliminal Emotional Faces Elicit Predominantly Right-Lateralized Amygdala Activation: A Systematic Meta-Analysis of fMRI Studies
Source: Front Neurosci. 2022 Jul 18;16:868366. doi: 10.3389/fnins.2022.868366 (PMC9339677; doi:10.3389/fnins.2022.868366)
Supplement: Supplementary file 4 [file Table_3.docx]

| **Supplementary Table S3:** Risk of bias assessment (AXIS tool) | | | | | | | | | | | | | | | | | | | | | | |
| --- | --- | --- | --- | --- | --- | --- | --- | --- | --- | --- | --- | --- | --- | --- | --- | --- | --- | --- | --- | --- | --- | --- |
|  | Killgore & Yurgelun-Todd (2004) | Nomura et al. (2004) | Phillips et al. (2004) | Etkin et al. (2004) | Liddell et al. (2005) | Williams et al. (2006) | Dannlowski et al. (2007a) | Dannlowski et al. (2007b) | Duan et al. (2010) | Suslow et al. (2010) | Mathiak et al. (2012) | Ottaviani et al. (2012) | Yang et al. (2012) | Dannlowski et al. (2013) | Prochnow et al. (2013) | Suslow et al. (2013) | Cui et al. (2014) | Pichon et al. (2016) | Redlich et al. (2017) | Victor et al. (2013) | Suslow et al. (2017) | Chen et al. (2017) |
| **Introduction** | | | | | | | | | | | | | | | | | | | | | | |
| 1. Were the aims/objectives of the study clear? | Yes | Yes | Yes | Yes | Yes | Yes | Yes | Yes | Yes | Yes | Yes | Yes | Yes | Yes | Yes | Yes | Yes | Yes | Yes | Yes | Yes | Yes |
| **Methods** | | | | | | | | | | | | | | | | | | | | | | |
| 2. Was the study design appropriate for the stated aim(s)? | Yes | Yes | Yes | Yes | Yes | Yes | Yes | Yes | Yes | Yes | Yes | Yes | Yes | Yes | Yes | Yes | Yes | Yes | Yes | Yes | Yes | Yes |
| 3. Was the sample size justified? | No | No | No | Yes | No | No | Yes | No | No | Yes | No | Yes | No | Yes | Yes | Yes | No | No | Yes | Yes | No | No |
| 4. Was the target/reference population clearly defined? (Is it clear who the research was about?) | Yes | Yes | Yes | Yes | Yes | Yes | Yes | Yes | Yes | Yes | Yes | Yes | Yes | Yes | Yes | Yes | Yes | Yes | Yes | Yes | Yes | Yes |
| 5. Was the sample frame taken from an appropriate  population base so that it closely represented the  target/reference population under investigation? | Yes | Yes | Yes | Yes | Yes | Yes | Yes | Yes | Yes | Yes | No | Yes | Yes | Yes | Yes | Yes | Yes | Yes | Yes | Yes | Yes | Yes |
| 6. Was the selection process likely to select subjects/participants that were representative of the target/reference population under investigation? | Yes | Yes | ND | ND | Yes | Yes | Yes | Yes | Yes | Yes | No | Yes | Yes | Yes | Yes | Yes | Yes | Yes | Yes | Yes | Yes | Yes |
| 7. Were measures undertaken to address and categorize non-responders? | Yes | Yes | Yes | Yes | Yes | Yes | Yes | Yes | Yes | Yes | Yes | Yes | Yes | Yes | Yes | Yes | ND | Yes | ND | ND | ND | Yes |
| 8. Were the risk factor and outcome variables measured appropriate to the aims of the study? | Yes | Yes | Yes | Yes | Yes | Yes | Yes | Yes | Yes | Yes | Yes | Yes | Yes | Yes | Yes | Yes | Yes | Yes | Yes | Yes | Yes | Yes |
| 9. Were the risk factor and outcome variables measured correctly using instruments/ measurements that had been trialled, piloted or published previously? | Yes | Yes | Yes | Yes | Yes | Yes | Yes | Yes | Yes | Yes | Yes | Yes | Yes | Yes | Yes | Yes | Yes | Yes | Yes | Yes | Yes | Yes |
| 10. Is it clear what was used to determined statistical significance and/or precision estimates? (e.g., p values, CIs) | Yes | Yes | Yes | Yes | Yes | Yes | Yes | Yes | Yes | Yes | Yes | Yes | Yes | Yes | Yes | Yes | Yes | Yes | Yes | Yes | Yes | Yes |
| 11. Were the methods (including statistical methods) sufficiently described to enable them to be repeated? | Yes | Yes | Yes | Yes | Yes | Yes | Yes | Yes | Yes | Yes | Yes | Yes | Yes | Yes | Yes | Yes | Yes | Yes | Yes | Yes | Yes | Yes |
| **Results** | | | | | | | | | | | | | | | | | | | | | | |
| 12. Were the basic data adequately described? | Yes | Yes | Yes | Yes | Yes | Yes | Yes | Yes | Yes | Yes | Yes | Yes | Yes | Yes | Yes | Yes | No | Yes | Yes | Yes | Yes | Yes |
| 13. Does the response rate raise concerns about nonresponse bias? | No | No | No | No | No | No | No | No | No | No | No | No | No | No | No | No | No | No | No | No | No | No |
| 14. If appropriate, was information about nonresponders described? | ND | yes | ND | Yes | ND | ND | Yes | Yes | ND | Yes | Yes | Yes | Yes | Yes | Yes | Yes | ND | ND | ND | ND | ND | Yes |
| 15. Were the results internally consistent? | Yes | Yes | Yes | Yes | Yes | Yes | Yes | Yes | Yes | Yes | Yes | Yes | Yes | Yes | Yes | Yes | Yes | Yes | Yes | Yes | Yes | Yes |
| 16. Were the results for the analyses described in the methods, presented? | Yes | Yes | Yes | Yes | Yes | Yes | Yes | Yes | Yes | Yes | Yes | Yes | Yes | Yes | Yes | Yes | Yes | Yes | Yes | Yes | Yes | Yes |
| **Discussion** | | | | | | | | | | | | | | | | | | | | | | |
| 17. Were the authors’ discussions and conclusions justified by the results? | Yes | Yes | Yes | Yes | Yes | Yes | Yes | Yes | Yes | Yes | Yes | Yes | Yes | Yes | Yes | Yes | Yes | Yes | Yes | Yes | Yes | Yes |
| 18. Were the limitations of the study discussed? | Yes | Yes | Yes | Yes | Yes | Yes | Yes | Yes | No | Yes | Yes | Yes | Yes | Yes | Yes | No | Yes | Yes | Yes | Yes | Yes | Yes |
| **Others** | | | | | | | | | | | | | | | | | | | | | | |
| 19. Were there any funding sources or conflicts of  interest that may affect the authors’ interpretation of  the results? | No | No | No | No | No | No | No | No | No | No | No | No | No | No | No | No | No | No | No | No | No | No |
| 20. Was ethical approval or consent of participants attained? | Yes | Yes | Yes | Yes | Yes | Yes | Yes | Yes | Yes | Yes | Yes | ND | Yes | Yes | Yes | Yes | Yes | Yes | Yes | Yes | Yes | Yes |
| Not described (ND)  Downes, M.J.; Brennan, M.L.; Williams, H.C.; Dean, R.S. Development of a critical appraisal tool to assess the quality of cross-sectional studies (AXIS). BMJ Open 2016, 6,  e011458, doi:10.1136/bmjopen-2016-011458. | | | | | | | | | | | | | | | | | | | | | | |
