# Supplementary material for: Subliminal Emotional Faces Elicit Predominantly Right-Lateralized Amygdala Activation: A Systematic Meta-Analysis of fMRI Studies
Source: Front Neurosci. 2022 Jul 18;16:868366. doi: 10.3389/fnins.2022.868366 (PMC9339677; doi:10.3389/fnins.2022.868366)
Supplement: Supplementary file 5 [file Table_4.docx]

| \| **Supplementary Table S4:** Contrasts in reviewed studies \| \| \| \| \| --- \| --- \| --- \| --- \| \| **Study name** \| **Authors** \| **Year** \| **Contrasts** \| \| [Activation of the amygdala and anterior cingulate during nonconscious processing of sad versus happy faces](https://www.sciencedirect.com/science/article/pii/S1053811904000072) \| Killgore & Yurgelun-Todd \| 2004 \| Masked happy faces vs neutral faces; masked sad faces vs neutral faces; masked happy/sad faces vs neutral faces \| \| [Functional association of the amygdala and ventral prefrontal cortex during cognitive evaluation of facial expressions primed by masked angry faces: an event-related fMRI study](https://www.sciencedirect.com/science/article/pii/S1053811903005706) \| Nomura et al. \| 2004 \| Masked angry faces vs neutral faces; masked angry faces vs white blank screen. \| \| Differential neural responses to overt and covert presentations of facial expressions of fear and disgust \| Phillips et al. \| 2004 \| Masked fearful faces vs neutral faces; masked disgusted faces vs neutral faces \| \| Individual differences in trait anxiety predict the response of the basolateral amygdala to unconsciously processed fearful faces. \| Etkin et al. \| 2004 \| Masked fearful faces vs neutral faces \| \| [A direct brainstem–amygdala–cortical ‘alarm’ system for subliminal signals of fear](https://www.sciencedirect.com/science/article/pii/S1053811904004768) \| Liddell et al. \| 2005 \| Masked fearful faces vs neutral faces \| \| [Amygdala–prefrontal dissociation of subliminal and supraliminal fear](https://doi.org/10.1002/hbm.20208) \| Williams et al. \| 2006 \| Masked fearful faces vs neutral faces \| \| [Amygdala reactivity predicts automatic negative evaluations for facial emotions](https://www.sciencedirect.com/science/article/pii/S0925492706000977) \| Dannlowski et al. \| 2007a \| Masked negative faces vs neutral faces \| \| Amygdala reactivity to masked negative faces is associated with automatic judgmental bias in major depression: a 3 T fMRI study \| Dannlowski et al. \| 2007b \| Masked sad faces vs a gray rectangle; masked angry faces vs a gray rectangle; masked happy faces vs a gray rectangle; masked sad faces vs neutral faces; masked angry faces vs neutral faces \| \| [Neural mechanism of unconscious perception of surprised facial expression](https://www.sciencedirect.com/science/article/pii/S1053811910004155) \| Duan et al. \| 2010 \| Masked surprised faces vs neutral faces; masked happy faces vs neutral faces \| \| [Automatic mood-congruent amygdala responses to masked facial expressions in major depression](https://www.sciencedirect.com/science/article/pii/S0006322309008981) \| Suslow et al. \| 2010 \| Masked happy faces vs neutral faces; and masked sad faces vs neutral faces \| \| [Lateralization of amygdala activation in fMRI may depend on phase-encoding polarity](https://link.springer.com/article/10.1007/s10334-011-0285-4) \| Mathiak et al. \| 2012 \| Masked fearful faces vs neutral faces \| \| [Amygdala responses to masked and low spatial frequency fearful faces: a preliminary fMRI study in panic disorder](https://www.sciencedirect.com/science/article/pii/S0925492711003957) \| Ottaviani et al. \| 2012 \| Masked fearful faces vs neutral faces \| \| [The amygdala is involved in affective priming effect for fearful faces](https://www.sciencedirect.com/science/article/pii/S027826261200067X) \| Yang et al. \| 2012 \| Masked fearful faces vs neutral faces \| \| [Childhood maltreatment is associated with an automatic negative emotion processing bias in the amygdala](https://pubmed.ncbi.nlm.nih.gov/22696400/) \| Dannlowski et al. \| 2013 \| Masked sad faces vs neutral faces; masked happy faces vs masked neutral faces \| \| [Processing of subliminal facial expressions of emotion: a behavioral and fMRI study](https://www.tandfonline.com/doi/full/10.1080/17470919.2013.812536?scroll=top&needAccess=true) \| Prochnow et al. \| 2013 \| Masked happy/sad/angry faces vs supraliminal \| \| [Neural correlates of affective priming effects based on masked facial emotion: An fMRI study](https://www.sciencedirect.com/science/article/abs/pii/S0925492712002454?via%3Dihub) \| Suslow et al. \| 2013 \| Masked sad faces vs neutral faces; masked happy faces vs masked neutral faces \| \| Trait emotional suppression is associated with increased activation of the rostral anterior cingulate cortex in response to masked angry faces. \| Cui et al. \| 2014 \| Masked angry faces vs neutral faces \| \| [Influence of Temporal Expectations on Response Priming by Subliminal Faces](https://www.ncbi.nlm.nih.gov/pmc/articles/PMC5072568/) \| Pichon et al. \| 2016 \| Masked fearful faces vs neutral faces \| \| [Effects of electroconvulsive therapy on amygdala function in major depression - a longitudinal functional magnetic resonance imaging study](https://pubmed.ncbi.nlm.nih.gov/28397635/) \| Redlich et al. \| 2017 \| Masked sad faces vs neutral faces \| \| [Sex Differences in Neural Responses to Subliminal Sad and Happy Faces in Healthy Individuals: Implications for Depression](https://www.ncbi.nlm.nih.gov/pmc/articles/PMC5120615/) \| Victor et al. \| 2017 \| Masked happy faces vs masked neutral faces; male vs female \| \| Brain response to masked and unmasked facial emotions as a function of implicit and explicit personality self-concept of extraversion. \| Suslow et al. \| 2017 \| Masked disgusted faces vs neutral faces; masked happy faces vs masked neutral faces \| \| Mismatch negativity (MMN) stands at the crossroads between explicit and implicit emotional processing \| Chen et al. \| 2017 \| Masked fearful faces vs masked neutral faces \| |
| --- | --- | --- | --- | --- | --- | --- | --- | --- | --- | --- | --- | --- | --- | --- | --- | --- | --- | --- | --- | --- | --- | --- | --- | --- | --- | --- | --- | --- | --- | --- | --- | --- | --- | --- | --- | --- | --- | --- | --- | --- | --- | --- | --- | --- | --- | --- | --- | --- | --- | --- | --- | --- | --- | --- | --- | --- | --- | --- | --- | --- | --- | --- | --- | --- | --- | --- | --- | --- | --- | --- | --- | --- | --- | --- | --- | --- | --- | --- | --- | --- | --- | --- | --- | --- | --- | --- | --- | --- | --- | --- | --- | --- | --- | --- | --- | --- |
